# Variations of Soybean Meal and Corn Mixed Substrates in Physicochemical Characteristics and Microbiota During Two-Stage Solid-State Fermentation

**DOI:** 10.3389/fmicb.2021.688839

**Published:** 2021-08-17

**Authors:** Weifa Su, Zipeng Jiang, Lihong Hao, Wentao Li, Tao Gong, Yu Zhang, Shuai Du, Cheng Wang, Zeqing Lu, Mingliang Jin, Yizhen Wang

**Affiliations:** National Engineering Laboratory of Biological Feed Safety and Pollution Prevention and Control, Key Laboratory of Molecular Nutrition, Ministry of Education, Key Laboratory of Animal Nutrition and Feed, Ministry of Agriculture, Key Laboratory of Animal Nutrition and Feed Science of Zhejiang Province, Institute of Feed Science, Zhejiang University, Hangzhou, China

**Keywords:** corn byproducts, two-stage solid-state fermentation, nutritional value, microbiota, *in vitro* digestibility

## Abstract

Corn germ meal (CGM) and corn gluten feed (CGF) are the two main corn byproducts (CBs) obtained from corn starch extraction. Due to their high fiber content, low protein content, and severe imbalance of amino acid, CBs are unable to be fully utilized by animals. In this study, the effect of microorganism, proteases, temperature, solid–liquid ratio, and time on nutritional properties of CB mixture feed (CMF) was investigated with the single-factor method and the response surface method to improve the nutritional quality and utilization of CBs. Fermentation with *Pichia kudriavzevii*, *Lactobacillus plantarum*, and neutral protease notably improved the nutritional properties of CMF under the fermentation conditions of 37°C, solid–liquid ratio (1.2:1 g/ml), and 72 h. After two-stage solid-stage fermentation, the crude protein (CP) and trichloroacetic acid-soluble protein (TCA-SP) in fermented CMF (FCMF) were increased (*p* < 0.05) by 14.28% and 25.53%, respectively. The *in vitro* digestibility of CP and total amino acids of FCMF were significantly improved to 78.53% and 74.94%, respectively. In addition, fermentation degraded fiber and provided more organic acids in the CMF. Multiple physicochemical analyses combined with high-throughput sequencing were performed to reveal the dynamic changes that occur during a two-stage solid-state fermentation process. Generally, *Ascomycota* became the predominant members of the community of the first-stage of fermentation, and after 36 h of anaerobic fermentation, *Paenibacillus* spp., *Pantoea* spp., and *Lactobacillales* were predominant. All of these processes increased the bacterial abundance and lactic acid content (*p* < 0.00). Our results suggest that two-stage solid-state fermentation with *Pichia kudriavzevii*, *Lactobacillus plantarum*, and protease can efficiently improve protein quality and nutrient utilization of CMF.

## Introduction

The competition for food between humans and livestock has become a topic with great concern in the past few years. The shortage of feed resources led to a sharp rise in the prices of conventional feed ingredients, such as corn, soybean meal, fish meal, etc. In order to reduce feed costs, less-expensive, alternative agricultural and industrial byproducts are increasingly included in livestock diets (Li et al., 2018). Corn is one of the most important cash crops in the world. Global corn production exceeded one billion tons per year, and the corn-processing industry produced large quantities of byproducts (Stein et al., 2016).

Corn germ meal (CGM) and corn gluten feed (CGF) are two main by-products obtained from corn starch extraction by wet milling (Figure 2B). CGM, the remaining portion of corn germ after oil removal, contains 30% protein, 18% starch, 12% cellulose, 2% ash, and 0.7% fat (Nielsen et al., 1979). CGF is produced by combining concentrated steepwater with the fiber during wet milling separation process, which typically contains 60% fiber and 20% protein. The low solubility and unbalanced AA composition of protein limit the application of CBs in the feed industry (Anderson et al., 2012). Solid-state fermentation in feedstock processing is an efficient biotechnological approach to improve nutritional value and increase nutrient bioavailability (Decimo et al., 2017; Wang et al., 2018a). *Bacillus subtilis*, *Pichia kudriavzevii*, and *Lactobacillus plantarum* were widely used SSF strains (Ni et al., 2017). *Bacillus subtilis* can degrade large molecular proteins to produce small peptides in SSF due to their capacity to secrete extracellular protease (Soares et al., 2005). Yeast (such as *Pichia kudriavzevii* and *Saccharomyces cerevisiae*) is most commonly used for agricultural byproduct SSF to obtain single-cell proteins (S) due to their low nutritional requirements, rapid growth, and high protein content (Hezarjaribi et al., 2016; Deng et al., 2020). The organic acids produced by *Lactobacillus plantarum*, mainly lactic acid, can reduce the pH of the fermentation system and inhibit the proliferation of some pathogenic bacteria and fungi (Missotten et al., 2015). In recent years, there have been many reports on the use of single bacteria to SSF agricultural byproducts, and a few study on the use of the abovementioned bacteria pairwise-combination fermentation to compare the fermentation effect. Our previous research shows that solid fermented soybean meal–corn mixture feed and distilled dried grain with soluble *Bacillus subtilis* and *Lactobacillus plantarum* can effectively improve their nutritional value and increase the content of small peptides and lactic acid (Shi et al., 2017; Wang et al., 2018b; Zhang et al., 2018; Zong et al., 2020). In addition, protease is widely used in SSF to degrade large molecular proteins to produce small peptides (Bedrosian and Kung, 2019). We hypothesized that using protease instead of extracellular protease-producing *Bacillus subtilis* to ferment with *Lactobacillus plantarum* and *Pichia kudriavzevii* would have a better fermentation effect.

In this study, a solid-state fermented feed system containing CBs, corn, soybean meal, and wheat bran was performed to gain a sufficient usage of CBs. To degrade fibers and produce digestible protein, *Bacillus subtilis*, *Lactobacillus plantarum*, and *Pichia kudriavzevii* were used for pairwise fermentation of CMF and reinforced by adding protease. Fermentation time, liquid ratio, and temperature would be traced to find better fermentation conditions and form a fermentation strategy.

## Materials and Methods

### Microorganisms and Enzymes

*Bacillus subtilis* ZJU12-1 (CGMCC No: 12825) and *Lactobacillus plantarum* CWLP (CGMCC No: 1.510) were obtained from Chinese traditional pickled vegetables. *Pichia kudriavzevii* PKWF was obtained from grains of a Chinese local distiller. *Bacillus subtilis* ZJU12-1, *Lactobacillus plantarum* CWLP, and *Pichia kudriavzevii* PKWF were maintained on Luria broth (LB), de Man, Rogosa, and Sharp (MRS), and yeast extract peptone dextrose (YPD) plates preserved at 4°C. Neutral protease was from *Bacillus* spp. (P3111; Sigma-Aldrich Corp.). CMF contains 20% CGM, 30% CGF, 30% corn, 15% soybean meal, and 5% wheat bran, which were obtained from the Cofine Bio-tech Co., Ltd. (Jiaxing, China).

### Preparation of Fermented Mixed Substrates

The fermentation substrates were set as follows to obtain the optimal fermentation combination: *B. subtilis* ZJU12-1 + *P. kudriavzevii* PKWF, *P. kudriavzevii* PKWF + *L. plantarum* CWLP, *B. subtilis* ZJU12-1 + *L. plantarum* CWLP, *P. kudriavzevii* PKWF + neutral protease, *L. plantarum* CWLP + neutral protease, and *L. plantarum* CWLP + *P. kudriavzevii* PKWF + neutral protease. Before fermentation, *B. subtilis* ZJU12-1 was cultured in LB liquid medium at 37°C for 12 h. *L. plantarum* CWLP was cultured in MRS liquid medium at 37°C for 18 h. *P. kudriavzevii* PKWF was cultured in YPD liquid medium at 37°C for 12 h. The 100 g CMF was mixed and placed in a 500-ml Erlenmeyer flask, and sterile water was added to achieve a solid–liquid ratio (1.5:1 g/ml). The wet mixed CMF was inoculated with microbes (7.0 log cfu/g) or protease (50 U/g) according to the fermentation combinations listed above. Then the flask was covered with a membrane (aerobic condition) and fermented at 37°C. The control group of CMF was treated without different bacteria and neutral protease, and other fermentation conditions were consistent. Moist samples (approximately 100 g) at 0, 36, and 72 h were collected to determine the numbers of microorganisms and microbial metabolites, and for 16S rRNA and ITS gene high-throughput sequencing, and the remaining samples were treated at 105°C for 30 min to prevent continuous fermentation. Then, the samples were dried at 65°C for 24 h, cooled, and ground, and subjected to physicochemical analysis, SDS-PAGE.

### Optimization of Fermentation Conditions

Based on the restriction of the fermentation conditions of SSF in actual scale production in China, the solid–liquid ratios (1.8:1, 1.5:1, and 1.2:1 g/ml), fermentation temperatures (27°C, 32°C, and 37°C), and fermentation times (24, 48, and 72 h) were investigated with response surface analysis to optimize the parameters of the optimal fermentation, and the Design Expert software (Version 8.0.6, Stat-Ease Inc., Minneapolis, MN, United States) was used for the regression and graphical analysis of the experimental data obtained.

*M* = *a*0 + *a*1 × *A* + *a*2 × *B* + *a*3 × *C* + *a*4 × *A*^2^ + *a*5 × *B*^2^ + *a*6 × *C*^2^ + *a*7 × *A* × *B* + *a*8 × *A* × *C* + *a*9 × *B* × *C*. *M* is the predicted response; *a*0 is the intercept term; *a*1, *a*2, and *a*3 are the linear coefficients; *a*4, *a*5, and *a*6 are the squared coefficients; and *a*7, *a*8, and *a*9 are the interaction coefficients.

To further study the effect of anaerobic condition on CMF fermentation quality, we used the two-stage SSF method. Briefly, the two-stage SSF process includes two stages; at the first stage, CMF was used as the main fermented substrate, which was inoculated with an effective combination of *Pichia kudriavzevii* and neutral protease at aerobic fermentation for 36 h; then at the second stage, *Lactobacillus plantarum* was inoculated into the first stage-fermented substrate at anaerobic fermentation for 36 h.

### Chemical Analyses

The dried samples of CMF were ground, sieved through a 1-mm sieve, and then the DM, CP, AA, EE, CF, NDF, and ADF were analyzed as described by AOAC (2005). Determination of TCA-SP in samples was performed using the method proposed by Ovissipour et al. (2009).

### Microorganisms and Microbial Metabolites

The pH and microbial counts were analyzed by the method of Wang et al. (2018b) with minor modifications. In brief, 5 g of wet samples was dissolved in 45 ml of sterile water and placed on a shaker at 150 rpm for 20 min. The pH of the supernatant was measured with a pH meter (Mettler Toledo, Switzerland). The samples were diluted 10-fold with sterile water for microbial counts. The viable count of *B. subtilis* was counted after culturing on LB agar for 24 h at 37°C. The viable count of *L. plantarum* was counted after culturing on MRS agar for 48 h at 37°C. The viable count of *P. kudriavzevii* was counted after culturing on YPD agar for 48 h at 37°C.

The concentration of organic acids (acetic acid, propionic acid, butyric acid, and lactic acid) in each sample was separated and quantified using a gas chromatograph (GC; GC−14B, Shimadzu, Japan; capillary column 30 m × 0.32 mm × 0.25 μm film thickness/VARIAN CP−3800, Varian, Palo Alto, CA, United States) as described by Franklin et al. (2002). In brief, the samples (1 g) were thawed and suspended in 2 ml of distilled water in a screw−capped tube. After being vortexed, each sample was centrifuged (12,000 × *g*) at 4°C for 10 min. The supernatant (1 ml) was transferred into a 2-ml centrifuge tube and mixed with 0.2 ml of metaphosphoric acid and kept at 4°C for 30 min. The mixtures were then centrifuged (12,000 × *g*) again at 4°C for 10 min. Aliquots of the supernatant (1 μl) were analyzed by GC.

### *In vitro* Digestibility

*In vitro* two-stage enzymatic hydrolysis process was performed by the method of Sakamoto et al. (2008) with minor modifications. In short, CMF or FCMF (2 g) was added to a 150-ml Erlenmeyer flask, containing 50 ml, 10,000 U/ml of pepsin (activity: 3,000 U/mg, Sigma) solution (0.05 mol/L KCl-HCl buffer, pH 2.0), and incubated on a shaker at 37°C, 100 rpm for 5 h. The pH of the mixture was then adjusted to 6.8 with 1 mol/L NaOH and 1 mol/L HCl, and 150 mg of trypsin (activity: 250 U/mg, Sigma) was added to the mixture and incubated on a shaker at 37°C, 100 rpm for 5 h. After digestion, 5 ml of 20% sulfosalicylic acid was added to the mixture and settled for 30 min. The digested slurry samples were centrifuged at 3,000 × *g* for 15 min. Then the precipitate, washed with doubled-distilled water for several times and collected, dried at 105°C, was used to analyze the content of CP and AA. *In vitro* CP (AA) digestibility (%) = [original CP (AA) amount − residual CP (AA) amount]/original CP (AA) amount × 100%.

### Microscopic Inspection

Changes in the physical properties of the substrates before and after fermentation were examined by SEM according to the protocol of the Electronic Microscopy Center of Zhejiang University. The microstructures of CMF and FCMF were observed using a field-emission scanning electron microscope (KYKY-EM3200, China) at ×100, ×1,000, and ×3,000 magnifications.

### Sodium Dodecyl Sulfate-Polyacrylamide Gel Electrophoresis

The proteins in CMF and FCMF were extracted using the procedure described by Faurobert (1997). The gel running conditions were chosen according to the report of Meinlschmidt et al. (2016). In brief, 12% polyacrylamide-separating gels were used for electrophoresis. Approximately 5 g of the protein sample was placed in each well, and the sample was separated at 55 mV for 200 min. The gel was stained with Coomassie Brilliant Blue (CBB) R-250 (Bio-Rad, United States) for 60 min and destained with 7% acetic acid.

### DNA Extraction and Illumina MiSeq Sequencing, and Metabolic Function Prediction

Total microbial genomic DNA, including bacterial and fungal genomic DNA, was extracted from the 18 samples using the E.Z.N.A soil DNA kit (Omega Bio-Tek, Norcross, GA, United States). A NanoDrop 2000 UV-vis spectrophotometer (Thermo Scientific, Wilmington, DE, United States) and 1% agarose gel electrophoresis were used to analyze DNA content and quality.

The V3–V4 gene regions of the bacterial 16S rRNA gene were amplified with primers 338F (5′-ACTCCTACGGGAGGCAGCAG-3=) and 806R (5′-GGACTACHVGGGTWTCTAAT-3=). The primer sequences for the ITS2 region of the fungal ITS gene were ITS1F (5′-CTTGGTCATTTAGAGGAAGTAA) and ITS2R (5′-GCTGCGTTCTTCATCGATGC). PCR was conducted as follows: 3 min of denaturation at 95°C; 27 cycles of 30 s at 95°C, 30 s of annealing at 55°C, and 45 s of elongation at 72°C; and a final extension at 72°C for 10 min. The AxyPrep DNA gel extraction kit (Axygen Biosciences, Union City, CA, United States) and QuantiFluor-ST instrument (Promega, United States) were used to further extract, purify, and quantify the PCR products. The MiSeq platform (Shanghai Majorbio Biopharm Technology Co., Ltd.) was used to describe the bacterial community based on the gene segment from the V3–V4 and ITS2 portion of the rRNA gene. Subsequently, raw illumina FASTQ files were demultiplexed, quality filtered, and analyzed using Quantitative Insights into Microbial Ecology (QIIME v1.9.1). Raw fastq files were quality filtered by Trichromatic and merged by FLASH. Operational taxonomic unit (OTUs) were clustered with 97% similarity cutoff using UPARSE (version 7.1). The taxonomy of each 16S rRNA gene sequence was analyzed using the RDP Classifier algorithm^1^ against the Greengenes 16S rRNA database using a confidence threshold of 70%. The assembled MiSeq sequences were submitted to the NCBI’s Sequence Read Archive (SRA BioProject no. PRJNA730509) for open access. Estimates of diversity values for these samples using the Chao1, Shannon, and Simpson indexes for diversity estimation were calculated by rarefaction analysis. Good’s coverage analysis was also performed. PCA and cluster analysis with the Ward method were conducted using the web server tool METAGENassist based on unweighted UniFrac distances. The main differentially abundant genera were selected by the LEfSe method^2^. To predict metabolic genes during the process, PICRUSt (see text footnote 2) was applied to obtain a functional profile from the 16S rRNA data. Prior to metagenome prediction, the OTUs of 16S rRNA sequences were analyzed using PICRUSt. PICRUSt and KEGG were used to obtain functions for the genes that were predicted to be present in the samples and to assign the genes into metabolic pathways. Fungal communities were analyzed and classified by the FUNGuild online tool^3^.

### Statistical and Bioinformatics Analysis

All assay data were analyzed using SPSS 20.0 software (SAS Inc., Chicago, IL, United States). One-way ANOVA and Duncan test were used to determine the difference between the mean values, and data are expressed as mean value ± standard deviation (SD). The differences between the means of the treatments were considered significant at *p* < 0.05. The heatmap package of R (R Core Team, 2014) was applied to generate heat maps of genera and L3-predicted microbial gene functions. Bar plots were generated in GraphPad Prism 8 (San Diego, CA, United States). Multiple testing corrections of distinguished species and predicted metabolic functions during fermentation were employed using Welch’s test and the Benjamini–Hochberg false-discovery rate (FDR) method for statistical analysis of metagenomic profiles (STAMP version 2.1.3).

## Results

### Selection of the Strain and Enzyme

Based on the results of the single-factor experiment (Table 1), CMF fermented with *P. kudriavzevii* PKWF, *L. plantarum* CWLP, and neutral protease (CMFPLN) has the highest content of CP, TCA-SP, and EE compared with the other fermentation combinations, which were 9.5%, 22.1%, and 38.1% higher than that of CMF, respectively. Furthermore, CF of CMFPLN is significantly lower than that of CMF (*p* < 0.05). These results indicate that the process of fermentation with *P. kudriavzevii* PKWF, *L. plantarum* CWLP, and neutral protease significantly improved the nutritional composition of CMF. In addition, CMFPLN has a more viable count of *P. kudriavzevii* PKWF and *L. plantarum* CWLP. The results above all suggest that CMFPLN may have the best fermentation potential.

**TABLE 1:** Nutrient composition and microorganisms of corn byproducts mixture feed (CMF) and CMF fermented with different microbes and neutral protease (as air-dry basis).

Items	CMF	CMFBP	CMFPL	CMFBL	CMFPN	CMFLN	CMFPLN
DM, %	90.33 ± 0.40	91.46 ± 0.48	91.08 ± 0.42	90.84 ± 0.56	90.80 ± 0.36	91.96 ± 0.22	91.04 ± 0.17
EE, %	2.23 ± 0.04e	2.51 ± 0.08d	2.59 ± 0.03cd	2.72 ± 0.06c	2.88 ± 0.08b	2.36 ± 0.08e	3.08 ± 0.14a
CP, %	22.27 ± 0.35d	23.60 ± 0.40b	23.42 ± 0.48bc	22.85 ± 0.25c	23.08 ± 0.14bc	23.29 ± 0.28bc	24.39 ± 0.24a
TCA-SP, %	37.45 ± 0.47e	44.22 ± 0.31bc	42.78 ± 0.27d	43.65 ± 0.39c	44.00 ± 0.29c	44.72 ± 0.24b	45.74 ± 0.13a
CF, %	6.11 ± 0.32a	5.33 ± 0.39bc	5.51 ± 0.42b	5.18 ± 0.14bc	5.37 ± 0.32*b*c	5.12 ± 0.10bc	4.91 ± 0.16c
pH	6.40 ± 0.03a	6.22 ± 0.10a	4.67 ± 0.09b	4.69 ± 0.17b	6.21 ± 0.12a	4.55 ± 0.07b	4.52 ± 0.18b
Microorganism, × 10^7^ CFU/g							
*Bacillus subtilis* ZJU12-1	–	82.00 ± 5.13	–	5.33 ± 8.19	–	–	–
*Pichia kudriavzevii* PKWF	–	1.80 ± 0.61	2.10 ± 0.44	–	1.71 ± 0.61	–	2.73 ± 0.65
*Lactobacillus plantarum* CWLP	–	–	16.67 ± 4.51	14.67 ± 4.51	–	16.00 ± 0.44	23.00 ± 6.25

*CMFBP, CMF fermented with *B. subtilis* ZJU12-1 and *P. kudriavzevii* PKWF; CMFPL, CMF fermented with *P. kudriavzevii* PKWF and *L. plantarum* CWLP; CMFBL, CMF fermented with *B. subtilis* ZJU12-1 and *L. plantarum* CWLP; CMFPN, CMF fermented with *P. kudriavzevii* PKWF and neutral protease; CMFLN, CMF fermented with *L. plantarum* CWLP and neutral protease; CMFPLN, CMF fermented with *P. kudriavzevii* PKWF, *L. plantarum* CWLP, and neutral protease. Values are mean ± SD, *n* = 3. Means followed with different superscript letters (a, b) within each line are significantly different (*p* < 0.05).*

### Optimal Fermentation Conditions of Corn Byproduct Mixture Feed Fermented With *Pichia kudriavzevii* PKWF, *Lactobacillus plantarum* CWLP, and Neutral Protease

**FIGURE 1:**
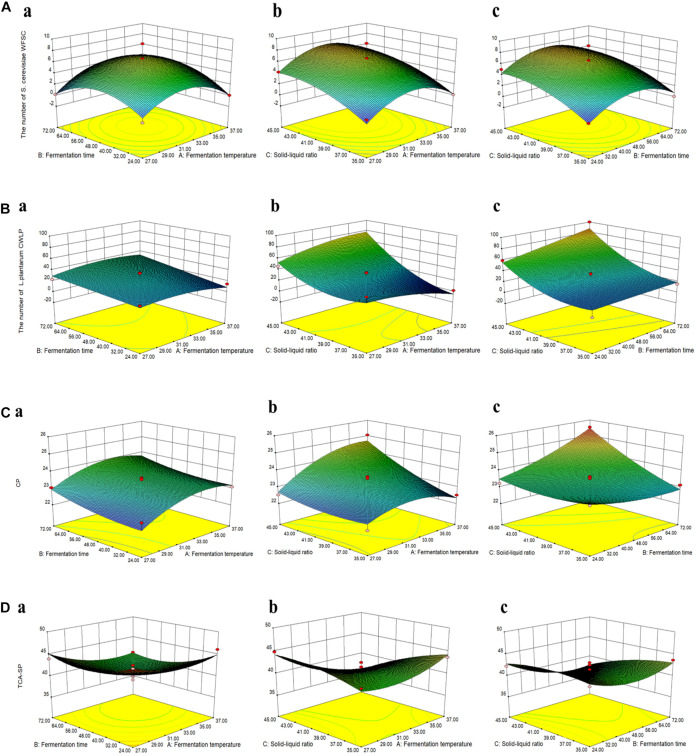
The response surface and contour plots showing the interactive effects of fermentation conditions on the viable count of *Pichia kudriavzevii* PKWF **(A)**, the viable count of *Lactobacillus plantarum* CWLP **(B)**, crude protein (CP) **(C)**, and trichloroacetic acid-soluble protein (TCA-SP) **(D)** of corn byproduct mixture feed fermented with *P. kudriavzevii* PKWF, *L. plantarum* CWLP, and neutral protease (CMFPLN) [**(a)** fermentation time and fermentation temperature; **(b)** solid–liquid ratio and fermentation temperature; **(c)** solid–liquid ratio and fermentation time].

Box–Behnken design (BBD) was employed to optimize three variables: fermentation temperature (A), fermentation time (B), and solid–liquid ratio (C) of CMFPLN. Based on the response surface results (Figure 1) and multiple regression analysis of the experimental data, the following second-order polynomial equation between the viable count of *P. kudriavzevii* PKWF, *L. plantarum* CWLP, and the three variables during fermentation were found: the viable count of *P. kudriavzevii* PKWF = 6.56 + 0.27 × *A* + 0.38 × *B* + 2.20 × *C* + 0.64 × *A* × *B* + 0.025 × *A* × *C* − 0.015 × *B* × *C* − 3.08 × *A*^2^ − 2.72 × *B*^2^ − 1.31 × *C*^2^, the viable count of *L. plantarum* CWLP = 28.80 − 1.3 × *A* + 8.89 × *B* + 27.36 × *C* + 3.38 × *A* × *B* + 13.83 × *A* × *C* + 7.10 × *B* × *C* − 6.55 × *A*^2^ − 0.13 × *B*^2^ + 15.42 × *C*^2^. Similarly, second-order polynomial equation between the content of CP, TCA-SP, and the three variables during fermentation were found: the content of CP = 23.41 + 0.47 × *A* + 0.34 × *B* + 0.65 × *C* − 0.046 × *A* × *B* + 0.52 × *A* × *C* + 0.66 × *B* × *C* − 0.60 × *A*^2^ + 0.20 × *B*^2^ − 0.22 × *C*^2^, the content of TCA-SP = 40.63 − 1.45 × *A* − 1.35 × *B* − 1.46 × *C* − 0.59 × *A* × *B* − 2.76 × *A* × *C* − 1.14 × *B* × *C* + 1.73 × *A*^2^ + 2.06 × *B*^2^ − 0.91 × *C*^2^.

**FIGURE 2:**
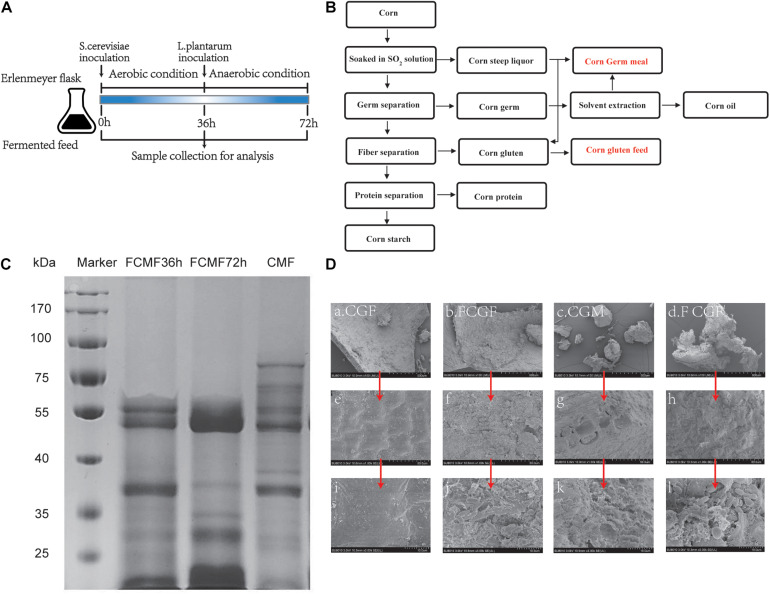
Experimental design, electrophoresis, and scanning electron microscopy (SEM) image of corn byproduct mixture feed (CMF) and fermented CMF (FCMF). **(A)** Experimental design. **(B)** Simplified process of corn wet milling for starch production and its byproducts. **(C)** Sodium dodecyl sulfate-polyacrylamide gel electrophoresis (SDS-PAGE) of FCMF at fermentation times of 0, 36, and 72 h. **(D)** SEM images of CMF and FCMF after 72 h of fermentation at ×100 **(a–d)**, ×1,000 **(e–h)**, and ×3,000 **(i–l)** fold magnifications.

By solving the regression equations above, the optimal condition of the three variables to obtain the maximum point of the model were calculated to be fermentation temperature at 32°C, fermentation time of 50 h, and a solid–liquid ratio of 1.2:1 g/ml, with the corresponding viable count of *P. kudriavzevii* PKWF at 7.50 × 10^7^ CFU/g. Similarly, under the optimal conditions of fermentation temperature at 37°C, fermentation time of 72 h, and a solid–liquid ratio of 1.2:1 g/ml, the viable count of *L. plantarum* CWLP reached the maximum value of 96.75 × 10^7^ CFU/g. The content of CP reached the maximum value of 25.86%, which was the same as the optimal conditions for the maximum viable count of *L. plantarum* CWLP. In addition, under the optimal conditions of fermentation temperature at 37°C, fermentation time of 24 h, and solid–liquid ratio of 1.8:1 g/ml, the content of TCA-SP reached the maximum value of 46.93%. In order to verify the optimization results, a verification experiment was conducted to show that the viable count of *P. kudriavzevii* PKWF, *L. plantarum* CWLP, the content of CP, and TCA-SP in their optimum conditions were 7.82 × 10^7^ CFU/g, 90.21 × 10^7^ CFU/g, 25.52%, and 46.21%, respectively, which indicated that the model was satisfactory and practicable.

### Two-Stage Solid-State Fermentation

Two-stage solid-stage fermentation was conducted and the process is presented in Figure 2A. The nutrient contents of CMF and CMF by two-stage solid-stage fermentation (FCMF) are presented in Table 2. Compared with CMF, the fermented CMF contained more CP, TCA-SP, and EE, which were augmented (*p* < 0.05) by approximately 14.28%, 25.33%, and 42.119%, respectively. Furthermore, the content of CF, ADF, and NDF were decreased (*p* < 0.05) by 29.10%, 10.43%, and 18.15%, respectively. In this study, the content of amylose and total starch were decreased dramatically after fermentation (*p* < 0.05). In addition, fermentation with inoculated microorganisms and neutral protease also affected the AA composition in CMF. In the present research, three indispensable AA (His, Ile, and Phe), two dispensable AA (Ser and Ala), and total AA significantly increased in FCMF compared with CMF. In addition, except for Asp, most AA showed an increase trend after fermentation. These results of the two-stage SSF were similar to the response surface analysis, which indicated that the oxygen was exhausted at the aerobic fermentation process and formed an anaerobic condition in the mixed substrates. This process is familiar with the two-stage SSF.

**TABLE 2:** Nutrient composition of CMF and fermented CMF (FCMF) (as air-dry basis).

Items	CMF	FCMF	Items	CMF	FCMF
DM, %	90.33 ± 0.02a	91.67 ± 0.02a	Indispensable AA,%		
EE, %	2.28 ± 0.14b	3.24 ± 0.11a	Arg	1.74 ± 0.02a	1.75 ± 0.01a
CP, %	22.33 ± 0.13b	25.52 ± 0.18a	His	0.59 ± 0.02b	0.66 ± 0.02a
TCA-SP, %	37.26 ± 1.00b	46.70 ± 0.78a	Ile	1.38 ± 0.01b	1.55 ± 0.06a
CF, %	6.70 ± 0.17a	4.05 ± 0.09b	Leu	0.74 ± 0.01a	0.76 ± 0.00a
ADF, %	10.64 ± 0.08a	9.53 ± 0.17b	Lys	0.74 ± 0.01a	0.76 ± 0.00a
NDF, %	28.71 ± 0.13a	23.50 ± 0.25b	Met	0.16 ± 0.01a	0.18 ± 0.01a
Total starch, %	14.49 ± 0.11a	13.96 ± 0.05b	Phe	0.75 ± 0.01b	0.83 ± 0.03a
Amylopectin, %	10.40 ± 0.14a	10.34 ± 0.07a	Thr	0.38 ± 0.01a	0.41 ± 0.01a
Amylose, %	4.09 ± 0.05a	3.62 ± 0.06b	Val	0.78 ± 0.01a	0.85 ± 0.05a
			Dispensable AA,%		
			Asp	1.74 ± 0.00a	1.71 ± 0.01a
			Ser	1.41 ± 0.01b	1.54 ± 0.00a
			Glu	4.61 ± 0.07a	4.76 ± 0.07a
			Gly	0.62 ± 0.01a	0.64 ± 0.00a
			Ala	1.32 ± 0.01b	1.48 ± 0.00a
			Cys	0.21 ± 0.01a	0.23 ± 0.00a
			Tyr	1.19 ± 0.03a	1.26 ± 0.05a
			Pro	1.41 ± 0.00a	1.37 ± 0.03a
			Total AA	19.61 ± 0.13b	20.67 ± 0.12a

*Values were mean ± SD, n = 3. Means followed with different superscript letters (a, b) within each line are significantly different (*p* < 0.05).*

### Microorganisms and Microbial Metabolites of Fermented Corn Byproduct Mixture Feed

To further evaluate the nutritional properties of FCMF, we determined the microorganisms and microbial metabolites after fermentation, and the results are presented in Table 3. After fermentation, the viable counts of *P. kudriavzevii* PKWF and *L. plantarum* CWLP in FCMF reached 6.95 × 10^7^ and 90.21 × 10^7^ CFU/g, respectively. The pH of CMF decreased from 6.42 to 4.54, which was mainly caused by organic acids produced by *L. plantarum* CWLP. The acetic acid, propionic acid, butyric acid, and lactic acid in FCMF were increased by 6.72, 20.83, 21.00, and 5.64 times, respectively.

**TABLE 3:** Microorganism and microbial metabolites of CMF and FCMF.

Items	CMF	FCMF
Organic acids, mg/100 g		
Acetic acid	22.07 ± 0.04b	148.22 ± 0.52a
Propionic acid	0.06 ± 0.01b	1.25 ± 0.03a
Butyric acid	0.18 ± 0.01b	3.78 ± 0.09a
Lactic acid	26.53 ± 0.08b	149.61 ± 0.09a
pH	6.42 ± 0.11a	4.54 ± 0.15b
Microorganism, 10^7^ CFU/g		
*P. kudriavzevii* PKWF	–	6.95 ± 0.60
*L. plantarum* WCLP	–	90.21 ± 8.40

*Values were mean ± SD, *n* = 3. Means followed with different superscript letters (a, b) within each line are significantly different (*p* < 0.05).*

### *In vitro* Digestibility of Fermented Corn Byproduct Mixture Feed

The results of the digestibility of CMF and FCMF are presented in Table 4. The *in vitro* digestibility of DM and CP in FCMF were notably improved by 18.98% and 16.62%. In addition, the *in vitro* digestibility of 14 AA, including 9 essential AAs (Arg, His, Ile, Leu, Met, Lys, Thr, Phe, and Val) and 5 dispensable AA (Ser, Glu, Ala, Tyr, and Cys) were significantly enhanced. Furthermore, after 72 h of fermentation, the digestibility of the average indispensable AA, average dispensable AA, and total AA were enormously improved by approximately 1.19, 1.17, and 1.13 times, respectively.

**TABLE 4:** *In vitro* crude protein (CP) and amino acid (AA) digestibility (%) of CMF and FCMF.

Items	CMF	FCMF
DM, %	48.89 ± 0.37b	58.17 ± 1.64a
CP, %	67.34 ± 0.70b	78.53 ± 0.51a
**Indispensable AA, %**		
Arg	64.50 ± 1.02b	73.71 ± 4.75a
His	66.11 ± 3.13b	79.37 ± 3.18a
Ile	52.58 ± 1.30b	66.33 ± 4.15a
Leu	57.25 ± 1.19b	64.92 ± 2.19a
Lys	50.67 ± 1.53b	60.67 ± 3.06a
Met	52.81 ± 1.51b	58.66 ± 0.60a
Phe	63.61 ± 0.54b	76.81 ± 1.92a
Thr	50.4 ± 1.48b	62.21 ± 1.15a
Val	51.86 ± 1.47b	65.91 ± 2.59a
**Dispensable AA, %**		
Asp	85.53 ± 0.19a	81.76 ± 1.59b
Ser	62.57 ± 1.76b	74.65 ± 1.10a
Glu	79.75 ± 1.51b	91.08 ± 1.94a
Gly	61.44 ± 0.99a	60.44 ± 1.97a
Ala	56.46 ± 1.13b	67.61 ± 1.96a
Cys	49.86 ± 1.29b	59.75 ± 4.25a
Tyr	55.35 ± 3.34b	63.55 ± 2.96a
Pro	66.91 ± 1.41a	68.83 ± 1.56a
Total AA, %	66.20 ± 0.31b	74.94 ± 1.27a

*Values were mean ± SD, *n* = 3. Means followed with different superscript letters (a, b) within each line are significantly different (*p* < 0.05).*

### Electrophoresis and Microscopic Observation

In the present study, the protein profiles of CMF were distributed in the range of 20–100 kDa (Figure 2C). However, in the fermentation with *P. kudriavzevii* PKWF, *L. plantarum* CWLP, and neutral protease for 72 h, the protein profile corresponding to multiple bands in the range of 55–100 kDa in FCMF were completely degraded. After 72 h of fermentation, the protein profile corresponding to multiple bands in the range of 30–50 kDa in FCMF were obviously degraded. In addition, the content of small peptides (<25 kDa) was significantly increased in FCMF compared with CMF.

Scanning electron microscopy (SEM) was applied to investigate the physical structures of CMF and FCMF. Figure 2D shows the surface images of CMF and FCMF at magnification factors of ×100, ×1,000, and ×3,000. After 72 h of fermentation, more fragmental structures were detected. At the same magnification factor, CGM and CGF in CMF had relatively large, compact, and smooth-faced structures, while the two main CBs in FCMF had smaller cracked structures and large holes. Besides, the surface of FCMF gathered more inoculated microbes.

### Changes in the Bacterial Community

**FIGURE 3:**
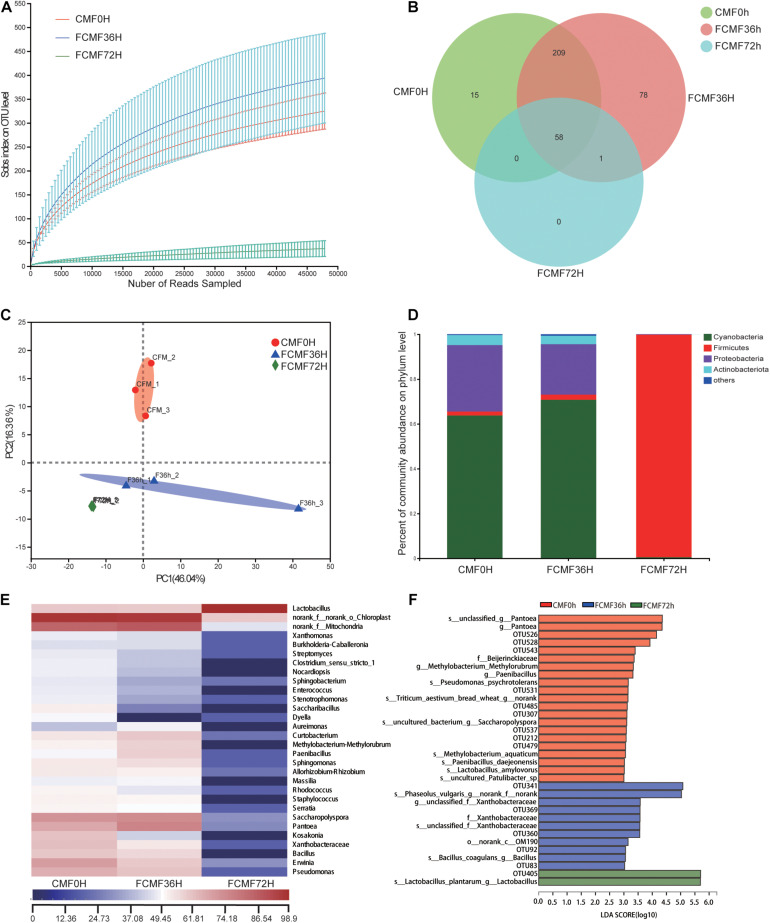
Microbial diversity and bacterial community structure during two-stage solid-state fermentation (SSF) (*n* = 3). **(A)** Observed operational taxonomic unit (OTU) line chart. **(B)** Venn diagram representing the common and unique OTUs found at each fermentation time point. **(C)** Principal-component (PC) analyses of samples conducted based on unweighted UniFrac distanced. **(D,E)** Phylum-level **(D)** and genus-level **(E)** compositions of the bacterial community in FCFM. **(F)** Linear discriminant analysis (LDA) effect size (LEFSe) histogram showing the LDA scores (>3.0) computed for features at the OTU level. Letters indicate the taxonomy of the bacteria: p, phylum, c, class; o, order; f, family; g, genus.

Overall, 229,415 high-quality sequences were collated. Additionally, the general 16S rRNA OTU numbers reached 798 based on 97% sequence similarity (Table 5). Combined with Good’s coverage index (99.6% ± 0.00%, data not shown), the results suggested that the samples exhibited abundant OTU coverage and that the sequencing depth was sufficient for analysis of the actual structure of the bacterial community during SSF. Figure 3A shows that the number of OTUs increased after the addition of *P. kudriavzevii* PKWF during the initial 36 h. In contrast, the number of OTUs decreased after the following 36 h of anaerobic fermentation. A Venn plot (Figure 3B) shows the common and unique OTUs in the groups. Fifty-eight OTUs as core genera were shared by all of the groups. The principal component analysis (PCA) plot (Figure 3C) shows that samples at 0, 36, and 72 h were well resolved and obviously distinct, while the OTU sample at 36 h exhibited fewer differences than others of the same group. In general, more than four bacterial phyla were found in all the samples (Figure 3D). In the CMF samples, *Cyanobacteria* and *Proteobacteria* accounted for 96.96% ± 0.10% of the sequences. However, as fermentation progressed, *Firmicutes* rapidly became the primary members of the community, accounting for approximately 99% of the sequences. With regard to the changes in bacterial community structure, the results at the gene level were similar to those at the phylum level (Figure 3E). Unfermented materials contained various native bacteria, including pathogens such as *Enterobacter* spp. and *Clostridium* spp. As the overall fermentation progressed, the predominant bacteria changed from *Cyanobacteria* and *Proteobacteria* to *Lactobacillus* spp.

**TABLE 5:** Characteristics of amplicon libraries in the bacteria community.

	Data for samples at time (h)			
Characteristic	CFM 0 h	FCMF 36 h	FCMF 72 h	Total no.
No. of sequences	59,862 ± 9,815	54,342 ± 5,696	57,857 ± 6080	229,415
No. of operational taxonomic units (OTUs)	405 ± 29^A^	406 ± 105^A^	41 ± 20^B^	798
Chao1 index	456 ± 64^A^	489 ± 69^A^	89 ± 21^B^	
Shannon index	1.69 ± 0.19^A^	1.75 ± 0.57^A^	0.06 ± 0.01^B^	
Simpson index	0.39 ± 0.11^A^	0.32 ± 0.11^A^	0.98 ± 0.01^B^	

*Means with different letters in each row differ at *p* < 0.05.*

Furthermore, the linear discriminant analysis (LDA) effect size (LEfSe) results showed significantly different taxonomy among different fermentation time points (Figure 3F). After 36 h of aerobic fermentation, the abundances of the genera *Bacillus* and *Xanthobacteraceae* increased significantly. After 36 h of anaerobic fermentation, *Paenibacillus* spp., *Pantoea* spp., and *Lactobacillales* were predominant.

### Changes in the Fungal Community

**FIGURE 4:**
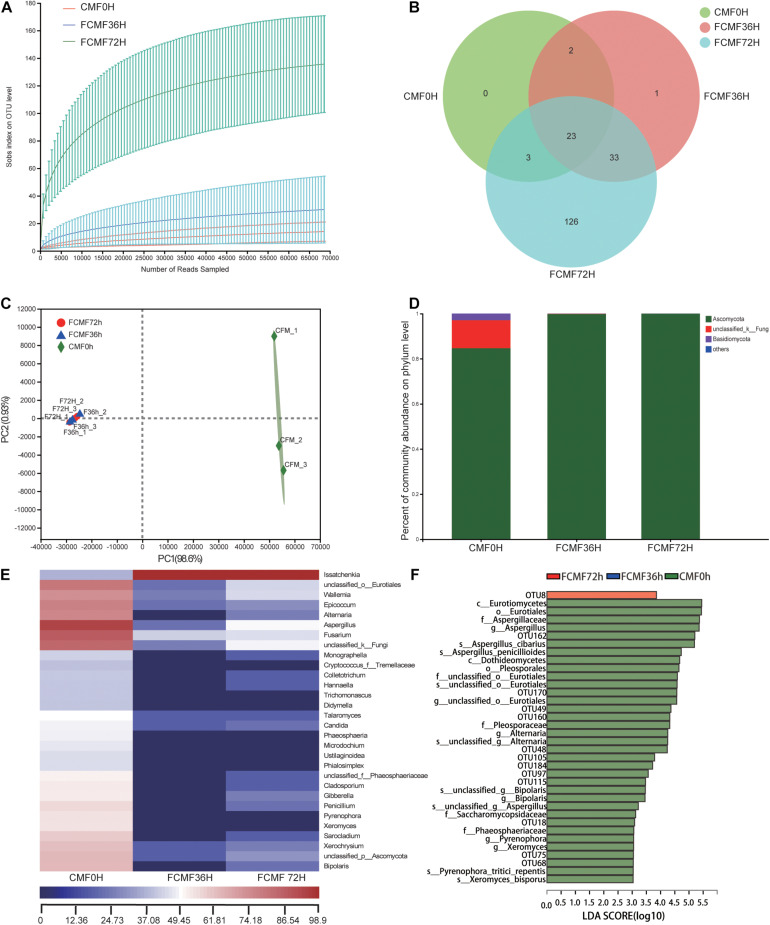
Microbial diversity and fungal community structure during two-stage SSF (*n* = 3). **(A)** Observed OTU line chart. **(B)** Venn diagram representing the common and unique OTUs found at each fermentation time point. **(C)** Principal component analyses (PCA) of samples conducted based on unweighted UniFrac distanced. **(D,E)** Phylum-level **(D)** and genus-level **(E)** compositions of the fungal community in FCFM. **(F)** LEFSe histogram showing the LDA scores (>3.0) computed for features at the OTU level. Letters indicate the taxonomy of the bacteria: p, phylum, c, class; o, order; f, family; g, genus.

Full of 644,350 high-quality sequences were collated byITS Genes Amplification, and the general operational taxonomic unit (OTU) numbers reached 547 based on 95% sequence similarity (Table 6). Combined with Good’s coverage index (99.7% ± 0.00%, data not shown), the results indicated that the samples exhibited abundant OTU coverage and that the sequencing depth was adequate for analysis of the actual structure of the fungal community during SSF. Figure 4A shows that the number of observed OTUs decreased after the addition of *P. kudriavzevii* PKWF during the first stage, then the number of observed OTUs decreased continuously after the following 36 h of anaerobic fermentation. A Venn plot (Figure 4B) shows the common and unique OTUs in the groups. Twenty-three OTUs as core genera were presented by all of the groups. The PCA plot (Figure 4C) illustrates that the samples between 0 and 36 h were significant resolved and obviously distinct, while the OTUs sample at 36 and 72 h exhibited fewer differences than those of the other samples. In general, more than three fungal phyla were found in all the samples (Figure 4D). In the CMF samples, *Ascomycota*, *unclassified_k_Fung*, and *Basidiomycota* occupied 97.96% ± 0.10% of the sequences. As fermentation progressed, *Ascomycota* became the predominant member of the community, accounting for approximately 99% of the sequences. When it comes to the changes in fungal community structure, the results at the gene level were similar to those at the phylum level (Figure 4E). Unfermented feed contained various native fungi, including pathogens such as *Colletotrichum* and *Fusarium*. As the overall fermentation progressed, the predominant fungi were kept unchanged with *Ascomycota*.

**TABLE 6:** Characteristics of amplicon libraries in the fungal community.

	Data for samples at time (h)			
Characteristic	CFM 0 h	FCMF 36 h	FCMF 72 h	Total no.
No. of sequences	69,999 ± 1,237	72,531 ± 2,410	72,252 ± 1,752	644350
No. of OTUs	136 ± 35^A^	31 ± 25^B^	14 ± 8^B^	547
Chao1 index	148 ± 28^A^	40 ± 27^B^	23 ± 62^B^	
Shannon index	2.26 ± 0.31^A^	0.10 ± 0.04^B^	0.81 ± 1.09^B^	
Simpson index	0.17 ± 0.05^B^	0.96 ± 0.01^A^	0.97 ± 0.00^A^	

*Means with different letters in each row differ at *p* < 0.05.*

Furthermore, the linear discriminant analysis (LDA) effect size (LEfSe) results showed significantly different taxonomy among different fermentation time points (Figure 4F). After 72 h of fermentation, the abundances of the OTU8 (*P. kudriavzevii*) increased significantly.

### Bacterial Metabolism of Fermented Mixed Substrates

**FIGURE 5:**
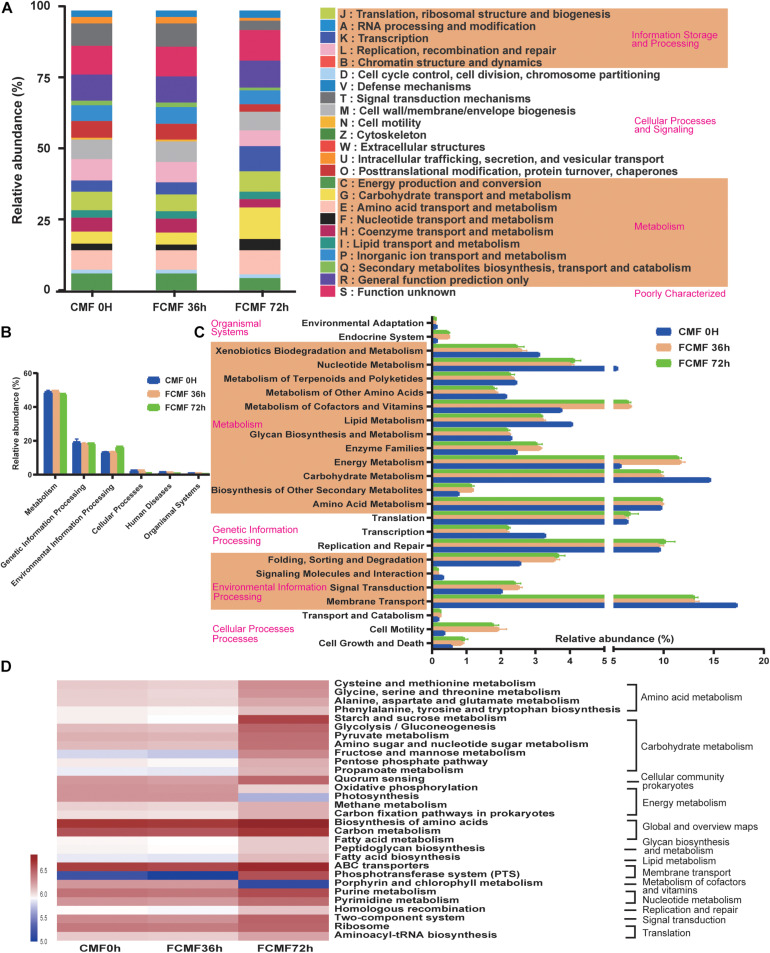
Dynamics of bacterial functional profiles during CMF fermentation process analyzed by PICRUSt (*n* = 3). **(A)** Clusters of Orthologous Groups of protein (COG) function classification. **(B)** Level 1 metabolic pathways. **(C)** Level 2 Kyoto Encyclopedia of Genes and Genomes (KEGG) ortholog functional predictions. **(D)** Level 3 KEGG ortholog functional predictions of the relative abundances of the top 30 metabolic functions.

The microbial metabolic functions presented in Figure 5 were obtained based on the Clusters of Orthologous Groups of proteins (COG) and Kyoto Encyclopedia of Genes and Genomes (KEGG) pathway database. Figure 5A shows the changes of COGs in three different fermentation time points, with the fermentation process, proteins related to metabolism functions (G,F) and information storage and processing functions (K) improved significantly; however, the functions of cellular processes and signaling (O,T,U,N,Z) decreased from 0 to 72 h. A majority of the predicted protein sequences ranged from 17.34% ± 0.02% to 0.05% ± 0.00% at the three time points among six different metabolic functions (Figure 5B), which represented different pathways (Figure 5C). Notably, carbohydrate metabolism, energy metabolism, and membrane transport accounted for more than 10% of the enriched pathways throughout the fermentation period. Furthermore, the sequences related to membrane transport, carbohydrate metabolism, energy metabolism, metabolism of cofactors and vitamins, nucleotide metabolism, and environmental adaptation were significantly enriched during intact fermentation process (*p* < 0.00).

At level 3 of the microbial gene functions of bacteria, some differences in efficiency were observed during SSF (Figure 5D). The abundance of a majority of the genes assigned to AA metabolism (cysteine and methionine metabolism, phenylalanine, tyrosine, and tryptophan biosynthesis) and carbohydrate metabolism (glycolysis/gluconeogenesis pyruvate metabolism, amino sugar and nucleotide sugar metabolism, fructose and mannose metabolism, pentose phosphate pathway, and propanoate metabolism) increased dramatically during the fermentation process (*p* < 0.05). Similarly, the genes associated with membrane transporter, such as ABC transporters and phosphotransferase system (PTS), were markedly enriched by the fermentation (*p* < 0.05). All these gene functions that improved were attributed to using *L. plantarum* during the second stage of fermentation. In contrast, the abundances of most genes related to glycan biosynthesis and metabolism and energy metabolism decreased with fermentation. Interestingly, the abundance of genes involved in global and overview maps (carbon metabolism) decreased during the aerobic fermentation period, while a considerable increase was observed following anaerobic fermentation.

**FIGURE 6:**
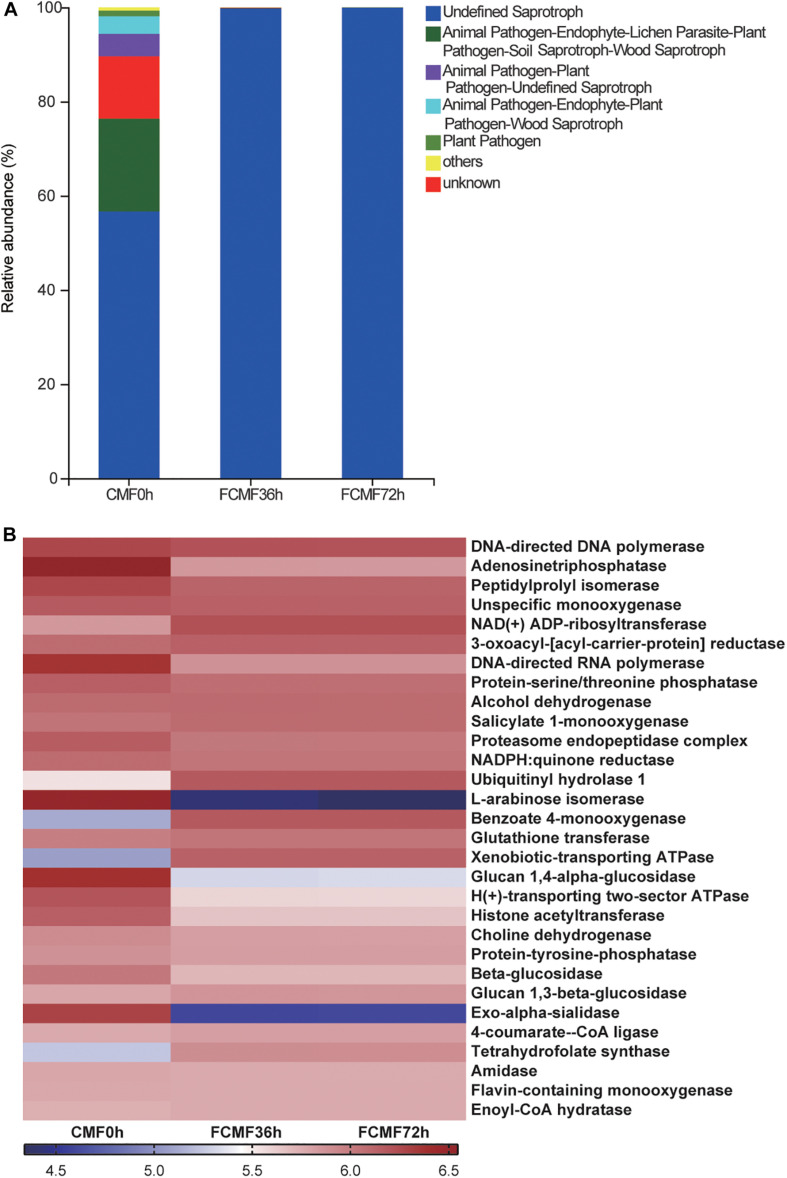
Dynamics of fungal functional profiles during CMF fermentation process analyzed by FUNGuild and PICRUSt (*n* = 3). **(A)** Variations in composition of fungal functional groups inferred by FUNGuild. **(B)** Level 3 KEGG ortholog functional predictions of the relative abundances of the top 30 enzymes.

As expected, the gene functions related to *P. kudriavzevii* were improved after the addition of *P. kudriavzevii* PKWF and reduced during the second-stage fermentation. The gene function prediction of the fungal community is presented in Figure 6. Fungal communities were analyzed by the FUNGuild online tool (see text footnote 3). In general, more than six fungal function groups were inferred by FUNGuild (Figure 6A). After adding *P. kudriavzevii*, the main fungal function group was Saprotroph, and it continued to the end of fermentation. In addition, the fungal functional groups inferred by FUNGuild, indicated to us that the unfermented CMF contain the animal pathogen and plant pathogen; in contrast, the fermented CMF could inhibit these pathogenic microbes and improve the safety and quality of CMF, and the level 3 KEGG ortholog functional predictions of the relative abundances exhibited several changes of key enzymes (Figure 6B). Adenosine triphosphatase, ubiquitinyl hydrolase 1, benzoate 4-monooxygenase, xenobiotic-transporting ATPase, and tetrahydrofolate synthase were significantly enriched during the intact fermentation process (*p* < 0.00), while peptidylprolyl isomerase, DNA-directed RNA polymerase, proteasome endopeptidase complex, L-arabinose isomerase, glucan 1,4-alpha-glucosidase, H(+)-transporting two-sector ATPase, histone acetyltransferase, and beta-glucosidase were decreased by adding *L. plantarum* CWLP.

## Discussion

In recent years, there have been many reports on the positive effects of bacteria or fungi (such as *P. kudriavzevii* and *L. plantarum*, etc.) and their metabolites in fermented feed. On the one hand, these microbes secrete a series of enzymes that effectively degrade anti-nutritional factors to improve the nutrient value of the feed materials (Arevalo-Villena et al., 2017). Besides, microorganisms and their functional metabolites, such as organic acids, cell wall polysaccharides, etc., can significantly improve the immune function of animals and inhibit the proliferation of pathogenic microorganisms, thereby maintaining the health of the animals (Fukuda et al., 2011; Zhu et al., 2014; Chen and Stappenbeck, 2019). *Bacillus* spp., *Lactobacillus* spp., and yeast were widely used in feed fermentation. However, the organic acids produced by *Lactobacillus* spp. would limit the activity of *Bacillus* spp. and, thus, inhibit its secretion of proteases (Wong and Chen, 1988). In the present study, we found that fermentation of *P. kudriavzevii* PKWF, *L. plantarum* CWLP, and neutral protease had the positive effect on improving CP and TCA-SP of CBs through single-factor experiments, which indicated that the role of *Bacillus* spp. in degrading proteins into TCA-SP could be replaced by neutral proteases.

Response surface analysis is an effective way to investigate the interaction between different factors during fermentation. Compared with the previous report (Li et al., 2019), the optimized fermentation conditions of soybean meal by response surface analysis were fermentation temperature (30°C), fermentation time (72 h), and solid–liquid ratio (1:3.5 g/ml); the protein hydrolysis of fermented soybean meal could reach to 10.05% by *Neurospora crassa* under the fermentation conditions. In our research, under the fermentation conditions of 32°C, solid–liquid ratio (1.2:1 g/ml), and 50 h, the proliferation of *P. kudriavzevii* PKWF reached its maximum. The rapid growth of *P. kudriavzevii* PKWF increased the consumption of oxygen, which provided an anaerobic environment for *L. plantarum* CWLP (Han et al., 2017). The viable count of *L. plantarum* CWLP reached its maximum value under the fermentation conditions of 37°C, solid–liquid ratio (1.2:1 g/ml), and 72 h. In addition, neutral protease may have the optimal enzyme activity to degrade macromolecular proteins into TCA-SP at 37°C. Accumulation of single-cell protein produced by microorganisms may lead to the increase in CP (Aggelopoulos et al., 2014; Mekoue Nguela et al., 2016), which reached its maximum under the fermentation conditions of 37°C, solid–liquid ratio (1.2:1 g/ml), and 72 h. In summary, we recommend the fermentation conditions of 37°C, solid–liquid ratio (1.2:1 g/ml), and 72 h for SSF of CMF.

The content of CP, TCA-SP, and AA in CMF was significantly increased after the fermentation in our research. In addition to the accumulation of single-cell proteins produced by microorganisms, the loss of DM (mainly carbohydrates) in the fermentation substrate may be another reason for the relative increase in the concentration of CP (Stokes and Gunness, 1946). TCA-SP consists of small peptides and free AA, most of which can be directly absorbed by the gastrointestinal tract (Gilbert et al., 2008). In addition, AA composition pattern changes during fermentation may be related to microbial protein synthesis and decomposition (Metges, 2000). Therefore, the increase in the content of TCA-SP and the change in AA composition can improve the nutritional value of CMF. Furthermore, the lignocellulosic components and amylose is poorly digested in the upper gut of monogastric animals (Regmi et al., 2011). CF, ADF, and amylose are effectively degraded after fermentation in this study, which might be due to the cellulase and amylase secreted by *L. plantarum* (Lee et al., 2019; Xu et al., 2020).

In contrast to CMF, FCMF exhibited small, cracked structures and large holes. The change in the surface structure of CMF after fermentation may be associated with extracellular enzymes (especially protease and carbohydrase) secreted during the process. The cracked and porous structure may provide increased access to enzymes for nutrient hydrolysis and may make the substrates considerably easier to utilize (Zheng et al., 2017), suggesting that FCMF had higher digestibility than CMF. Additionally, Tang et al. (2009) reported that smaller protein aggregates may result in a higher solubility. Zhao et al. (2015) found that soybean proteins with loose networks and diffuse structures have higher emulsification activity and solubility. Thus, the physicochemical properties of CMF may also have been affected by the changed microstructure in this study.

Digestibility is an essential method to evaluate the nutritional value of protein. The increase in CP and TCA-SP and the optimization of AA composition pattern in FCMF may be the main reasons for improving the *in vitro* digestibility of CP and AA. Some macromolecular proteins induce allergic reactions in humans and animals (Holzhauser et al., 2009; Wu et al., 2016). In the present study, CMF, fermented with *P. kudriavzevii* PKWF, *L. plantarum* CWLP, and neutral protease, contained less macromolecular proteins and more small peptides compared with CMF; the result was consistent with the previous report (Chi and Cho, 2016). In addition, the degradation of viscous-resistant starch and cellulose in FCMF leads to the exposure of internal proteins to the environment of pepsin and trypsin (Agama-Acevedo et al., 2005; Yang et al., 2010), which possibly contributes to the *in vitro* digestibility of CP and AA. Furthermore, the low pH value of FCMF is more effective to promote the function of pepsin (Mat et al., 2018).

High-throughput sequencing was first applied to analyze the changes in microbial community, including bacterial and fungal communities, structure, and metabolic functions during the fermentation process. The increase in OTU number during first-stage fermentation was because of the aerobic condition. In contrast, the second-stage fermentation process suggested that *L. plantarum* CWLP decreased OTU number, inhibited other bacteria and fungi, and became the dominant bacterium. The main phyla (*Cyanobacteria*, *Proteobacteria*, *and Firmicutes*) found in the present study were also obtained in some other studies related to SSF (Wang et al., 2020).

As fermentation progressed, *unclassified_k_Fung* and *Basidiomycota* became the predominant members of the community; *Ascomycota* were identified as the core genera during first stage of SSF. This type of fungi could consume oxygen in the first stage of fermentation, which may inhibit other pathogenic aerobic microorganisms. Meanwhile, *Paenibacillus* spp., *Pantoea* spp., and *Lactobacillales* spp. were predominant during the later 36 h, and members of the heat-tolerant genus *Paenibacillus* spp. are effective at degrading proteins and cellulose during SSF due to their strong hydrolytic abilities (de Gannes et al., 2013). *Lactobacillales* spp. is a mesophilic genus whose members generate acid products (Yuan et al., 2018). Thus, these dominant genera indicated a selected community categorized by typical large-molecule catabolism characteristics, achieved by the addition of the two inoculated microbes. The evolution of bacterial and fungal structure during the process demonstrated that the artificially added inoculated microbes not only increased the number of added microbes but also boosted some other functional microbes that could develop a form of symbiosis with the inoculated microbes.

The results of KEGG levels 1 to 3 gene function analysis were generally consistent. As fermentation progressed, the abundances of both carbohydrate metabolism and AA metabolism genes gradually increased. Metabolism of cellulose and hemicellulose can produce many compounds that support bacterial growth (Toledo et al., 2017). AAs are also an energy and carbon source for bacteria (Sanchez et al., 2017). These results indicated that the degradation of large carbohydrate and proteins resulted in increased levels of saccharides and AAs, which could be utilized by the microbiota in FCMF. The gene abundances of membrane transporter, ABC transporters, and phosphotransferase system (PTS) increased during fermentation. These metabolic functions were associated with compound production and membrane transport, suggesting the mechanism of enzyme synthesis by, and activity of, the core bacteria in FCMF. In contrast, glycan biosynthesis and metabolism, and energy metabolism were inhibited by the core genera, indicating that these genes may be involved in native bacterial gene functions of CMF. Although the addition of *L. plantarum* inhibited the growth of other CMF-native microbes, it did not decrease the abundances of enzyme families. Additionally, the differences in the abundances of genes involved in global and overview maps (carbon metabolism) between the aerobic and anaerobic stages suggested the different metabolic roles of *P. kudriavzevii* and *L. plantarum*.

Various bacteria and fungi were correlated with different metabolic pathways, revealing that multiple metabolic pathways were active during the two stages of the SSF. Two inoculated microbes were enriched in environmental information processing and cellular processes throughout fermentation. These metabolic functions allowed the microbe to grow, proliferate, and respond to the environment (Kausar et al., 2011). The results demonstrated the superior adaptation of the inoculated microbes in response to fermentation.

## Conclusion

In summary, two-stage SSF with *P. kudriavzevii* PKWF, *L. plantarum* CWLP, and neutral protease under the conditions of fermentation temperature of 37°C, fermentation time of 72 h, and solid-liquid ratio of 1.2:1 g/ml effectively improved protein digestibility in CMF through degrading macromolecular protein into TCA-SP, improving AA composition patterns and degrading lignocellulose to expose internal nutrients. Therefore, this study provides a novel method for improving the nutritional quality of CMF and provides a basis for demonstrating that the inoculated microbes dynamically change the physicochemical features, microbiota, and metabolic functions during the two-stage SSF, which could serve as a valuable resource for industrial feed-based practices and metabolomic research on SSF systems; besides, this study also provides a strategy for the utilization of CBs as feed materials. Further studies should focus on the use of additional enzymes and inoculation with other bacteria during fermentation to further reduce the ANF content of FCMF and produce various types of organic acids.

## Data Availability Statement

The original contributions presented in the study are included in the article/Supplementary Material, further inquiries can be directed to the corresponding authors.

## Author Contributions

WS: investigation, data curation, and writing-original draft. ZJ: investigation, software, and writing original draft. LH: visualization. WL: project administration. TG: resources. YZ: formal analysis. SD and CW: software and validation. ZL and MJ: conceptualization, supervision, and writing review and editing. YW: supervision, funding acquisition, methodology, software, and writing review and editing. All authors contributed to the article and approved the submitted version.

## Conflict of Interest

The authors declare that the research was conducted in the absence of any commercial or financial relationships that could be construed as a potential conflict of interest.

## Publisher’s Note

All claims expressed in this article are solely those of the authors and do not necessarily represent those of their affiliated organizations, or those of the publisher, the editors and the reviewers. Any product that may be evaluated in this article, or claim that may be made by its manufacturer, is not guaranteed or endorsed by the publisher.

## Footnotes

1
http://rdp.cme.msu.edu/

2
https://huttenhower.sph.harvard.edu/galaxy/

3
http://www.funguild.org/

## Abbreviations

SSF: solid-state fermentation
CGM: corn germ meal
CGF: corn gluten feed
CBs: corn byproducts
CMF: CBs mixture feed
FCMF: two-stage solid-state fermented CBs mixture feed
DM: dry matter
EE: ether extract
AA: amino acid
CP: crude protein
TCA-SP: trichloroacetic acid soluble protein
CF: crude fiber
ADF: acid detergent fiber
NDF: neutral detergent fiber
SDS-PAGE: sodium dodecyl sulfate-polyacrylamide gel electrophoresis.
