# Supplementary material for: Variations of Soybean Meal and Corn Mixed Substrates in Physicochemical Characteristics and Microbiota During Two-Stage Solid-State Fermentation
Source: Front Microbiol. 2021 Aug 17;12:688839. doi: 10.3389/fmicb.2021.688839 (PMC8416090; doi:10.3389/fmicb.2021.688839)
Supplement: Supplementary file 1 [file Data_Sheet_1.PDF]

BLAST ® » blastn suite » RID-MYFF7220014

BLAST Results

Questions/comments

Job title: Z1013-7

**RID**	MYFF7220014 (Expires on 09-03 14:38 pm)
**Query ID**	lcl|Query_55297
**Description**	None
**Molecule type**	dna
**Query Length**	1639
**Database Name**	nt
**Description**	Nucleotide collection (nt)
**Program**	BLASTN 2.10.1+

Graphic Summary

Distribution of the top 121 Blast Hits on 100 subject sequences

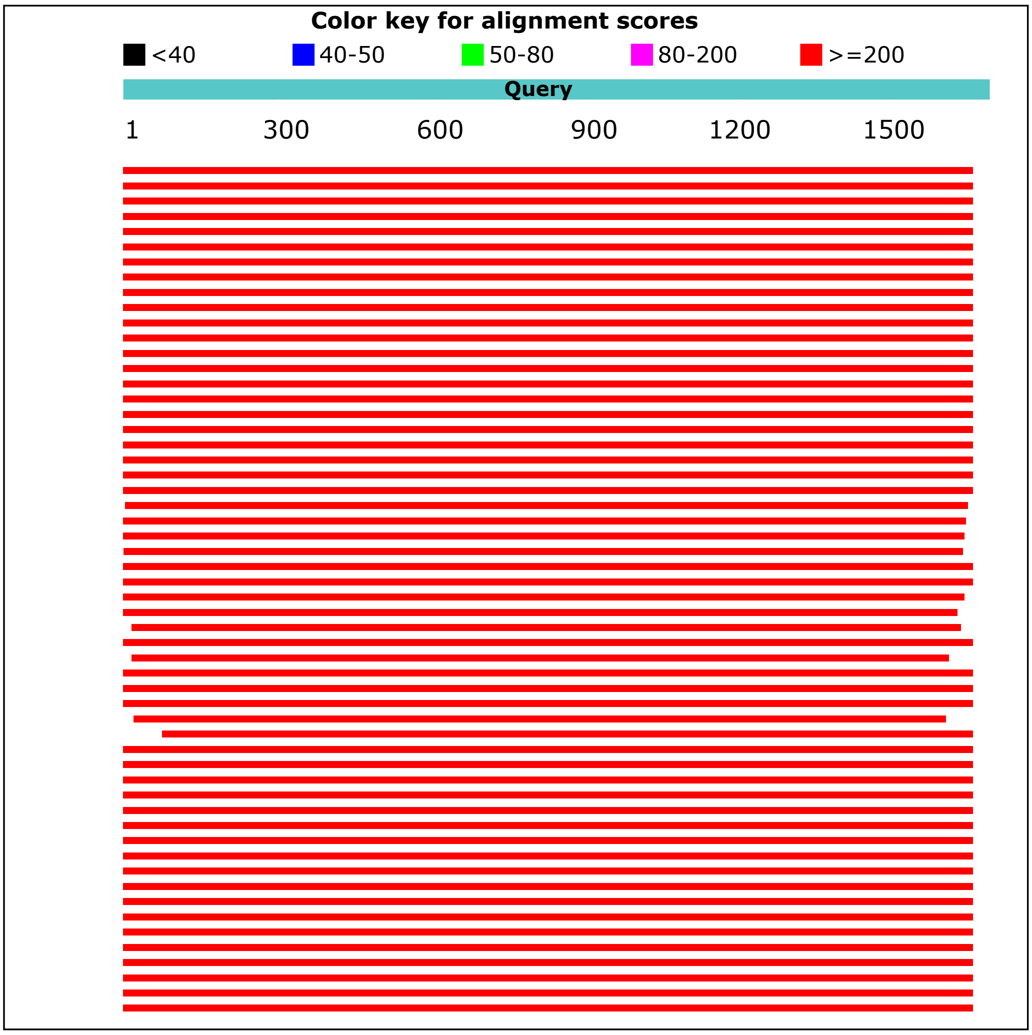

Descriptions

Sequences producing significant alignments:

Description	Max Score	Total Score	Query Cover	E value	Per. Ident	Accession
Uncultured Pichia clone m1 18S ribosomal RNA gene, partial sequence	3022	3022	100%	0.0	99.94%	HM151325.1
Uncultured Pichia clone p7 18S ribosomal RNA gene, partial sequence	3022	3022	100%	0.0	99.94%	HM151318.1
Pichia kudriavzevii 18S ribosomal RNA (C5L36_RDN18a), rRNA	3020	3020	99%	0.0	99.94%	XR_003834612.1
Pichia kudriavzevii strain CY902 chromosome III	3020	6040	99%	0.0	99.94%	CP039617.1
Pichia kudriavzevii strain CY902 chromosome II	3020	6040	99%	0.0	99.94%	CP039615.1
Pichia kudriavzevii strain CY902 chromosome I	3020	29974	99%	0.0	99.94%	CP039612.1
Pichia kudriavzevii NRRL Y-5396 18S rRNA gene, partial sequence; from TYPE material	3020	3020	99%	0.0	99.94%	NG_063274.1
Pichia kudriavzevii strain CBS573 chromosome 2, complete sequence	3020	3020	99%	0.0	99.94%	CP028774.1
Pichia kudriavzevii strain CBS573 chromosome 1, complete sequence	3020	6040	99%	0.0	99.94%	CP028773.1
Pichia kudriavzevii strain CBS5147 chromosome 2, complete sequence	3020	3020	99%	0.0	99.94%	CP028532.1
Pichia kudriavzevii strain CBS5147 chromosome 1, complete sequence	3020	3020	99%	0.0	99.94%	CP028531.1
Pichia kudriavzevii strain SJP chromosome 1	3020	14993	99%	0.0	99.94%	CP021088.1
Pichia cecembensis genomic DNA containing 18S ribosomal RNA gene, partial sequence region, strain CBS 10445	3020	3020	99%	0.0	99.94%	LT854929.1
Pichia kudriavzevii isolate EM12 18S ribosomal RNA gene, partial sequence	3020	3020	99%	0.0	99.94%	JF274497.1
Pichia kudriavzevii strain IPE100 18S ribosomal RNA gene, partial sequence	3020	3020	99%	0.0	99.94%	GQ166761.1
Pichia kudriavzevii strain NRRL Y-5396 18S ribosomal RNA gene, partial sequence	3020	3020	99%	0.0	99.94%	EF550360.1
Issatchenkia orientalis 18S ribosomal RNA gene, partial sequence	3020	3020	99%	0.0	99.94%	AY964117.1
Pichia kudriavzevii strain SJP chromosome 3	3016	3016	99%	0.0	99.88%	CP021090.1
Issatchenkia orientalis 18S ribosomal RNA gene, partial sequence	3016	3016	100%	0.0	99.88%	DQ438180.1
Pichia kudriavzevii strain SJP chromosome 5	3014	3014	99%	0.0	99.88%	CP021092.1
Saccharomyces sp. WW-W46 18S ribosomal RNA gene, partial sequence	3014	3014	99%	0.0	99.88%	DQ345283.2
Candida krusei small ribosomal subunit RNA gene sequence	3014	3014	99%	0.0	99.88%	M55528.1
Pichia kudriavzevii strain LSA 18S ribosomal RNA gene, partial sequence	3001	3001	99%	0.0	100.00%	KT025851.1
Issatchenkia orientalis strain N5 18S ribosomal RNA gene, partial sequence	2996	2996	99%	0.0	99.94%	AY218894.1

Description	Max Score	Total Score	Query Cover	E value	Per. Ident	Accession
Pichia kudriavzevii strain Y1 18S ribosomal RNA gene, partial sequence	2990	2990	98%	0.0	99.94%	MG321582.1
Pichia cecembensis isolate S-I 15 small subunit ribosomal RNA gene, partial sequence	2987	2987	98%	0.0	99.94%	MG460473.1
Issatchenkia orientalis gene for 18S rRNA, partial sequence	2987	2987	99%	0.0	99.51%	AB053239.2
Issatchenkia orientalis gene for 18S rRNA, partial sequence	2987	2987	99%	0.0	99.57%	AB160862.1
Pichia cecembensis isolate S-I F small subunit ribosomal RNA gene, partial sequence	2983	2983	98%	0.0	99.88%	MG460472.1
Pichia kudriavzevii strain 103 18S ribosomal RNA gene, partial sequence	2963	2963	98%	0.0	99.88%	KX397033.1
Pichia kudriavzevii strain Y6 18S ribosomal RNA gene, partial sequence	2953	2953	97%	0.0	100.00%	MG321587.1
Issatchenkia sp. NRRL Y-12824 18S ribosomal RNA gene, partial sequence	2922	2922	99%	0.0	98.84%	EF550384.1
Pichia kudriavzevii strain Y11 18S ribosomal RNA gene, partial sequence	2913	2913	96%	0.0	100.00%	MG321590.1
Pichia sp. 18S rRNA gene, strain IGC 4931	2898	2898	99%	0.0	98.60%	X96459.1
Candida pseudolambica strain NRRL Y-17318 18S ribosomal RNA gene, partial sequence	2896	2896	99%	0.0	98.54%	EF550373.1
Pichia jaroonii gene for 18S rRNA, partial sequence, strain: S-75	2894	2894	99%	0.0	98.54%	AB436770.1
Pichia kudriavzevii strain Y13 18S ribosomal RNA gene, partial sequence	2892	2892	95%	0.0	100.00%	MG321592.1
Pichia kudriavzevii strain CBS 5147 18S small subunit ribosomal RNA gene, partial sequence; internal transcribed spacer 1, 5.8S ribosomal RNA gene, and internal transcribed spacer 2, complete sequence; and 26S large subunit ribosomal RNA gene, partial sequence	2883	2883	95%	0.0	100.00%	MH545928.1
Candida pseudolambica gene for 18S rRNA, partial sequence	2874	2874	99%	0.0	98.29%	AB053238.2
Issatchenkia sp. NRRL Y-12827 18S ribosomal RNA gene, partial sequence	2870	2870	99%	0.0	98.29%	EF550383.1
Pichia exigua strain NRRL Y-10920 18S ribosomal RNA gene, partial sequence	2870	2870	99%	0.0	98.29%	EF550375.1
[Candida] phayaonensis genomic DNA containing 18S ribosomal RNA gene, partial sequence region, strain CBS 12319	2859	2859	99%	0.0	98.17%	LT854933.1
Pichia occidentalis NRRL Y-7552 18S rRNA gene, partial sequence; from TYPE material	2859	2859	99%	0.0	98.17%	NG_063277.1
Candida rugopelliculosa strain NRRL Y-17079 18S ribosomal RNA gene, partial sequence	2854	2854	99%	0.0	98.11%	EF550376.1
Pichia garciniae genomic DNA containing 18S ribosomal RNA gene, partial sequence region, strain CBS 10758	2850	2850	99%	0.0	98.05%	LT854927.1

Description	Max Score	Total Score	Query Cover	E value	Per. Ident	Accession
Pichia membranifaciens genomic DNA containing 18S ribosomal RNA gene, partial sequence region, strain CBS 1329	2850	2850	99%	0.0	98.05%	LT854920.1
Pichia sp. NRRL Y-27259 18S ribosomal RNA gene, partial sequence	2850	2850	99%	0.0	98.05%	EF550366.1
Issatchenkia scutulata var. exigua gene for 18S rRNA, partial sequence, strain:JCM 1829	2850	2850	99%	0.0	98.05%	AB053247.2
Pichia sp. 18S rRNA gene, strain IGC 4595	2850	2850	99%	0.0	98.05%	X96460.1
Pichia sp. IFO 1788 gene for 18S rRNA, partial sequence	2850	2850	99%	0.0	98.05%	AB019217.1
Pichia membranifaciens IFO 10215 18S rRNA gene, partial sequence; from TYPE material	2850	2850	99%	0.0	98.05%	NG_064813.1
P.membranaefaciens 18S rRNA gene, strain IGC 5012	2848	2848	99%	0.0	98.05%	X96453.1
Pichia membranifaciens strain NRRL Y-2026 18S ribosomal RNA gene, partial sequence	2844	2844	99%	0.0	97.99%	JQ698896.1
Uncultured Pichia clone m2 18S ribosomal RNA gene, partial sequence	2844	2844	99%	0.0	97.99%	HM151323.1
Issatchenkia sp. NRRL Y-12830 18S ribosomal RNA gene, partial sequence	2844	2844	99%	0.0	97.99%	EF550382.1
Pichia scutulata strain NRRL Y-7663 18S ribosomal RNA gene, partial sequence	2844	2844	99%	0.0	97.99%	EF550381.1
Pichia sp. NRRL YB-4149 18S ribosomal RNA gene, partial sequence	2844	2844	99%	0.0	97.99%	EF550362.1
P.membranaefaciens 18S rRNA gene, strain IGC 4275	2844	2844	99%	0.0	97.99%	X96451.1
Pichia jaroonii strain CB2 18S ribosomal RNA gene, partial sequence	2843	2843	99%	0.0	97.94%	HM009317.1
P.membranaefaciens 18S rRNA gene, strain IGC 5043	2843	2843	99%	0.0	97.99%	X96452.1
Pichia nakasei NRRL Y-7686 18S rRNA gene, partial sequence; from TYPE material	2841	2841	99%	0.0	97.93%	NG_063282.1
Pichia nakasei strain NRRL Y-7686 18S ribosomal RNA gene, partial sequence	2841	2841	99%	0.0	97.93%	EF550386.1
Pichia membranifaciens isolate DR4 18S ribosomal RNA gene, and internal transcribed spacer 1, complete sequence	2841	2841	99%	0.0	97.93%	AY251635.1
Issatchenkia occidentalis gene for 18S rRNA, partial sequence	2837	2837	99%	0.0	97.93%	AB053240.2
Pichia kluyveri NRRL Y-11519 18S rRNA gene, partial sequence; from TYPE material	2835	2835	99%	0.0	97.87%	NG_063285.1
Pichia kluyveri strain NRRL Y-11519 18S ribosomal RNA gene, partial sequence	2835	2835	99%	0.0	97.87%	EF550389.1
Pichia fermentans 18S ribosomal RNA gene, partial sequence	2835	2835	99%	0.0	97.87%	DQ489318.1
Candida sp. BG00-10-19-1-7-4 18S ribosomal RNA gene, partial sequence	2835	2835	99%	0.0	97.87%	AY242193.1

Description	Max Score	Total Score	Query Cover	E value	Per. Ident	Accession
Issatchenkia hanoiensis strain FN8S01 18S ribosomal RNA gene, partial sequence	2833	2833	100%	0.0	97.87%	FJ153095.1
Pichia membranifaciens strain NRRL Y-2026 18S ribosomal RNA gene, partial sequence	2833	2833	99%	0.0	97.81%	EF550365.1
Pichia barkeri NRRL Y-17350 18S rRNA gene, partial sequence; from TYPE material	2832	2832	99%	0.0	97.81%	NG_063281.1
Pichia barkeri strain NRRL Y-17350 18S ribosomal RNA gene, partial sequence	2832	2832	99%	0.0	97.81%	EF550385.1
P.membranaefaciens gene for 18S ribosomal RNA	2832	2832	99%	0.0	97.81%	X58055.1
Pichia sp. strain SM11UFAM small subunit ribosomal RNA gene, partial sequence; internal transcribed spacer 1, 5.8S ribosomal RNA gene, and internal transcribed spacer 2, complete sequence; and large subunit ribosomal RNA gene, partial sequence	2830	2830	100%	0.0	97.81%	MN268782.1
Pichia manshurica genomic DNA containing 18S ribosomal RNA gene, partial sequence region, strain CBS 209	2828	2828	99%	0.0	97.81%	LT854925.1
Pichia manshurica strain NRRL Y-17349 18S ribosomal RNA gene, partial sequence	2828	2828	99%	0.0	97.81%	EF550361.1
Pichia membranifaciens 18S ribosomal RNA gene, partial sequence	2828	2828	99%	0.0	97.81%	AY964118.1
Pichia manshurica isolate WI2 18S ribosomal RNA gene, and internal transcribed spacer 1, complete sequence	2828	2828	99%	0.0	97.81%	AY251637.1
Pichia manshurica IFO 10726 18S rRNA gene, partial sequence; from TYPE material	2828	2828	99%	0.0	97.81%	NG_064814.1
Pichia sporocuriosa NRRL Y-27347 18S rRNA gene, partial sequence; from TYPE material	2826	2826	99%	0.0	97.80%	NG_065546.1
P.membranaefaciens 18S rRNA gene, strain IGC 5003	2826	2826	99%	0.0	97.81%	X96455.1
Pichia pseudocactophila NRRL Y-17239 18S rRNA gene, partial sequence; from TYPE material	2824	2824	99%	0.0	97.75%	NG_063280.1
Pichia pseudocactophila strain NRRL Y-17239 18S ribosomal RNA gene, partial sequence	2824	2824	99%	0.0	97.75%	EF550380.1
Pichia cactophila NRRL Y-10963 18S rRNA gene, partial sequence; from TYPE material	2822	2822	99%	0.0	97.74%	NG_063279.1
Pichia deserticola NRRL Y-12918 18S rRNA gene, partial sequence; from TYPE material	2822	2822	99%	0.0	97.75%	NG_063275.1
Pichia cactophila strain NRRL Y-10963 18S ribosomal RNA gene, partial sequence	2822	2822	99%	0.0	97.74%	EF550379.1
Pichia deserticola strain NRRL Y-12918 18S ribosomal RNA gene, partial sequence	2822	2822	99%	0.0	97.75%	EF550364.1
Candida ethanolica strain NRRL Y-12615 18S ribosomal RNA gene, partial sequence	2822	2822	99%	0.0	97.75%	EF550363.1

Description	Max Score	Total Score	Query Cover	E value	Per. Ident	Accession
[Candida] rugopelliculosa JCM 1593 18S rRNA gene, partial sequence; from TYPE material	2822	2822	99%	0.0	97.68%	NG_063475.1
Candida ethanolica isolate EM2 18S ribosomal RNA gene, partial sequence	2819	2819	100%	0.0	97.69%	JF274496.1
Candida inconspicua strain NRRL Y-2029 18S ribosomal RNA gene, partial sequence	2819	2819	99%	0.0	97.68%	EF550378.1
Pichia sp. 18S rRNA gene, strain IGC 5047	2817	2817	99%	0.0	97.56%	X96461.1
Candida sp. BG02-7-20-001A-1-1 18S ribosomal RNA gene, partial sequence	2811	2811	99%	0.0	97.62%	AY520289.1
Pichia sp. 18S rRNA gene, strain IGC 5019	2811	2811	99%	0.0	97.56%	X96456.1
Pichia kluyveri strain SM12UFAM small subunit ribosomal RNA gene, partial sequence; internal transcribed spacer 1, 5.8S ribosomal RNA gene, and internal transcribed spacer 2, complete sequence; and large subunit ribosomal RNA gene, partial sequence	2808	2808	98%	0.0	97.91%	MN268784.1
Pichia chibodasensis NBRC 111569 18S rRNA gene, partial sequence; from TYPE material	2806	2806	99%	0.0	97.56%	NG_065112.1
[Candida] thaimueangensis genomic DNA containing 18S ribosomal RNA gene, partial sequence region, strain CBS 10360	2806	8848	99%	0.0	97.56%	LT854930.1
Pichia membranifaciens gene for 18S rRNA, partial sequence	2806	2806	99%	0.0	97.56%	AB053233.2
Pichia cephalocereana NRRL Y-17225 18S rRNA gene, partial sequence; from TYPE material	2802	2802	99%	0.0	97.45%	NG_063284.1
Pichia cephalocereana strain NRRL Y-17225 18S ribosomal RNA gene, partial sequence	2802	2802	99%	0.0	97.45%	EF550388.1

Alignments

Uncultured Pichia clone m1 18S ribosomal RNA gene, partial sequence
Sequence ID: **HM151325.1** Length: 1688 Number of Matches: 1
Range 1: 26 to 1665

Score	Expect	Identities	Gaps	Strand	Frame
3022 bits(1636)	0.0()	1639/1640(99%)	1/1640(0%)	Plus/Plus	

Features:

```
Query  1    CATTATACGGTG-AACTGCGAATGGCTCATTAAATCAGTTATCGTTTATTTGATAGTTCC  59
Sbjct  26   CATTATACGGTGAAACTGCGAATGGCTCATTAAATCAGTTATCGTTTATTTGATAGTTCC  85
Query  60   GTTCTACATGGATAACCGTGGAAAATCTAGAGCTAATACATGCGTAAAGCCCCGACTTCG  119
Sbjct  86   GTTCTACATGGATAACCGTGGAAAATCTAGAGCTAATACATGCGTAAAGCCCCGACTTCG  145
Query  120  GGAGGGGTGTATTTATTAGATAAAAAATCAATGCCCTCGGGCCTTTTGATGATTCATAAT  179
Sbjct  146  GGAGGGGTGTATTTATTAGATAAAAAATCAATGCCCTCGGGCCTTTTGATGATTCATAAT  205
Query  180  AACTTTTCGAAGCTCATGGCCTTGCGCCGGAGCTGGTTCATTCAAATTTCTGCCCTATCA  239
Sbjct  206  AACTTTTCGAAGCTCATGGCCTTGCGCCGGAGCTGGTTCATTCAAATTTCTGCCCTATCA  265
Query  240  ACTTTCGATGGTAGGATAGAGGCCTACCATGGTTTTCACGGGTAACGGGGAATAAGGGTT  299
Sbjct  266  ACTTTCGATGGTAGGATAGAGGCCTACCATGGTTTTCACGGGTAACGGGGAATAAGGGTT  325
Query  300  CGATTCCGGAGAGGGAGCCTGAGAAACGGCTACCACATCCAAGGAAGGCAGCAGGCGCGC  359
Sbjct  326  CGATTCCGGAGAGGGAGCCTGAGAAACGGCTACCACATCCAAGGAAGGCAGCAGGCGCGC  385
```

```
Query  360   AAATTACCCAATCCTGACACAGGGAGGTAGTGACAATATATAACGATACAGGGCCTTTGG  419
             ||||||||||||||||||||||||||||||||||||||||||||||||||||||||||||
Sbjct  386   AAATTACCCAATCCTGACACAGGGAGGTAGTGACAATATATAACGATACAGGGCCTTTGG  445

Query  420   TCTTGTAATTGGAATGAGTACAATGTAAATACCTTAACGAGGAACAATTGGAGGGCAAGT  479
             ||||||||||||||||||||||||||||||||||||||||||||||||||||||||||||
Sbjct  446   TCTTGTAATTGGAATGAGTACAATGTAAATACCTTAACGAGGAACAATTGGAGGGCAAGT  505

Query  480   CTGGTGCCAGCAGCCGCGGTAATTCCAGCTCCAATAGCGTATATTAAAGTTGTTGCAGTT  539
             ||||||||||||||||||||||||||||||||||||||||||||||||||||||||||||
Sbjct  506   CTGGTGCCAGCAGCCGCGGTAATTCCAGCTCCAATAGCGTATATTAAAGTTGTTGCAGTT  565

Query  540   AAAAAGCTCGTAGTTGAACTTTGGGCCTGGGCGGACGGTCTACCTATGGTAAGCACTGTT  599
             ||||||||||||||||||||||||||||||||||||||||||||||||||||||||||||
Sbjct  566   AAAAAGCTCGTAGTTGAACTTTGGGCCTGGGCGGACGGTCTACCTATGGTAAGCACTGTT  625

Query  600   GCGGCCGGGTCTTTCCTTCTGGCTAGCCCTCGGGCGAACCAGGACGATTACTTTGAGGAA  659
             ||||||||||||||||||||||||||||||||||||||||||||||||||||||||||||
Sbjct  626   GCGGCCGGGTCTTTCCTTCTGGCTAGCCCTCGGGCGAACCAGGACGATTACTTTGAGGAA  685

Query  660   ATTAGAGTGTTCAAAGCAGGCCTTTGCTCGGATATATTAGCATGGAATAATAGAATAGGA  719
             ||||||||||||||||||||||||||||||||||||||||||||||||||||||||||||
Sbjct  686   ATTAGAGTGTTCAAAGCAGGCCTTTGCTCGGATATATTAGCATGGAATAATAGAATAGGA  745

Query  720   CGCATGGTTCTATTTTGTTGGTTTCTAGGACCATCGTAATGATTAATAGGGACGGTCGGG  779
             ||||||||||||||||||||||||||||||||||||||||||||||||||||||||||||
Sbjct  746   CGCATGGTTCTATTTTGTTGGTTTCTAGGACCATCGTAATGATTAATAGGGACGGTCGGG  805

Query  780   GGCATCAGTATTCAGTCGTCAGAGGTGAAATTCTTGGATTGACTGAAGACTAACTACTGC  839
             ||||||||||||||||||||||||||||||||||||||||||||||||||||||||||||
Sbjct  806   GGCATCAGTATTCAGTCGTCAGAGGTGAAATTCTTGGATTGACTGAAGACTAACTACTGC  865

Query  840   GAAAGCATTTGCCAAGGACGTTTTCATTAATCAAGAACGAAAGTTAGGGGATCGAAGATG  899
             ||||||||||||||||||||||||||||||||||||||||||||||||||||||||||||
Sbjct  866   GAAAGCATTTGCCAAGGACGTTTTCATTAATCAAGAACGAAAGTTAGGGGATCGAAGATG  925

Query  900   ATCAGATACCGTCGTAGTCTTAACCATAAACTATGCCGACTAGGGATCGGGTGGTGCTAC  959
             ||||||||||||||||||||||||||||||||||||||||||||||||||||||||||||
Sbjct  926   ATCAGATACCGTCGTAGTCTTAACCATAAACTATGCCGACTAGGGATCGGGTGGTGCTAC  985

Query  960   TTTGCCCACTCGGCACCTTACGAGAAATCAAAGTTTTTGGGTTCTGGGGGGAGTATGGTC  1019
             ||||||||||||||||||||||||||||||||||||||||||||||||||||||||||||
Sbjct  986   TTTGCCCACTCGGCACCTTACGAGAAATCAAAGTTTTTGGGTTCTGGGGGGAGTATGGTC  1045

Query  1020  GCAAGGCTGAAACTTAAAGGAATTGACGGAAGGGCACCACCAGGAGTGGAGCCTGCGGCT  1079
             ||||||||||||||||||||||||||||||||||||||||||||||||||||||||||||
Sbjct  1046  GCAAGGCTGAAACTTAAAGGAATTGACGGAAGGGCACCACCAGGAGTGGAGCCTGCGGCT  1105

Query  1080  TAATTTGACTCAACACGGGGAAACTCACCAGGTCCAGACGTAATAAGGATTGACAAGTTA  1139
             ||||||||||||||||||||||||||||||||||||||||||||||||||||||||||||
Sbjct  1106  TAATTTGACTCAACACGGGGAAACTCACCAGGTCCAGACGTAATAAGGATTGACAAGTTA  1165

Query  1140  GAGACTTCTCTTGATCTTACGGGTGGTGGTGCATGGCCGTTTTTAGTCCTTGGAGTGATT  1199
             ||||||||||||||||||||||||||||||||||||||||||||||||||||||||||||
Sbjct  1166  GAGACTTCTCTTGATCTTACGGGTGGTGGTGCATGGCCGTTTTTAGTCCTTGGAGTGATT  1225

Query  1200  TGTCTGCTTAATTGCGATAACGGACGAGACCTTAACCTGCTAAATAGGGCTGCGAGCATC  1259
             ||||||||||||||||||||||||||||||||||||||||||||||||||||||||||||
Sbjct  1226  TGTCTGCTTAATTGCGATAACGGACGAGACCTTAACCTGCTAAATAGGGCTGCGAGCATC  1285

Query  1260  TGCTCGGGTGCTCTTCTTAGAGGGACTATGGGTATCAAACCCATGGAAGTTTGAGGCAAC  1319
             ||||||||||||||||||||||||||||||||||||||||||||||||||||||||||||
Sbjct  1286  TGCTCGGGTGCTCTTCTTAGAGGGACTATGGGTATCAAACCCATGGAAGTTTGAGGCAAC  1345

Query  1320  AACAGGTCTGTGATGCCCTTAGACGTTCTGGGCCGCACGCGCGCTACACTGACGGAGCCA  1379
             ||||||||||||||||||||||||||||||||||||||||||||||||||||||||||||
Sbjct  1346  AACAGGTCTGTGATGCCCTTAGACGTTCTGGGCCGCACGCGCGCTACACTGACGGAGCCA  1405

Query  1380  GCAAGTCCAACCTTGGTCGAGAGGCCCGGGTAATCTCGTGAAACTCCGTCGTGCTGGGGA  1439
             ||||||||||||||||||||||||||||||||||||||||||||||||||||||||||||
Sbjct  1406  GCAAGTCCAACCTTGGTCGAGAGGCCCGGGTAATCTCGTGAAACTCCGTCGTGCTGGGGA  1465

Query  1440  TAGAGCATTGTAATTTTTGCTCTTCAACGAGGAATTCCTAGTAAGCGCAAGTCATCAGCT  1499
             ||||||||||||||||||||||||||||||||||||||||||||||||||||||||||||
Sbjct  1466  TAGAGCATTGTAATTTTTGCTCTTCAACGAGGAATTCCTAGTAAGCGCAAGTCATCAGCT  1525

Query  1500  TGCGTTGATTACGTCCCTGCCCTTTGTACACACCGCCCGTCGCTACTACCGATTGAATGG  1559
             ||||||||||||||||||||||||||||||||||||||||||||||||||||||||||||
Sbjct  1526  TGCGTTGATTACGTCCCTGCCCTTTGTACACACCGCCCGTCGCTACTACCGATTGAATGG  1585

Query  1560  CTTAGTGAGGCTTCAAGATTGGCGCCGCGGGAGGGGCAACTTTCCCATGGGGCCGAGAAT  1619
             ||||||||||||||||||||||||||||||||||||||||||||||||||||||||||||
Sbjct  1586  CTTAGTGAGGCTTCAAGATTGGCGCCGCGGGAGGGGCAACTTTCCCATGGGGCCGAGAAT  1645

Query  1620  CTAGTCAAACTTGGTCATTA  1639
             ||||||||||||||||||||
Sbjct  1646  CTAGTCAAACTTGGTCATTA  1665
```

Uncultured Pichia clone p7 18S ribosomal RNA gene, partial sequence

Sequence ID: **HM151318.1** Length: 1684 Number of Matches: 1

Range 1: 21 to 1660

Score	Expect	Identities	Gaps	Strand	Frame
3022 bits(1636)	0.0()	1639/1640(99%)	1/1640(0%)	Plus/Plus	

Features:

```
Query  1     CATTATACGGTG-AACTGCGAATGGCTCATTAAATCAGTTATCGTTTATTTGATAGTTCC  59
             |||||||||||| |||||||||||||||||||||||||||||||||||||||||||||||
Sbjct  21    CATTATACGGTGAAACTGCGAATGGCTCATTAAATCAGTTATCGTTTATTTGATAGTTCC  80

Query  60    GTTCTACATGGATAACCGTGGAAAATCTAGAGCTAATACATGCGTAAAGCCCCGACTTCG  119
             ||||||||||||||||||||||||||||||||||||||||||||||||||||||||||||
Sbjct  81    GTTCTACATGGATAACCGTGGAAAATCTAGAGCTAATACATGCGTAAAGCCCCGACTTCG  140
```

```
Query  120   GGAGGGGTGTATTTATTAGATAAAAAATCAATGCCCTCGGGCCTTTTGATGATTCATAAT  179
             ||||||||||||||||||||||||||||||||||||||||||||||||||||||||||||
Sbjct  141   GGAGGGGTGTATTTATTAGATAAAAAATCAATGCCCTCGGGCCTTTTGATGATTCATAAT  200

Query  180   AACTTTTCGAAGCTCATGGCCTTGCGCCGGAGCTGGTTCATTCAAATTTCTGCCCTATCA  239
             ||||||||||||||||||||||||||||||||||||||||||||||||||||||||||||
Sbjct  201   AACTTTTCGAAGCTCATGGCCTTGCGCCGGAGCTGGTTCATTCAAATTTCTGCCCTATCA  260

Query  240   ACTTTCGATGGTAGGATAGAGGCCTACCATGGTTTTCACGGGTAACGGGGAATAAGGGTT  299
             ||||||||||||||||||||||||||||||||||||||||||||||||||||||||||||
Sbjct  261   ACTTTCGATGGTAGGATAGAGGCCTACCATGGTTTTCACGGGTAACGGGGAATAAGGGTT  320

Query  300   CGATTCCGGAGAGGGAGCCTGAGAAACGGCTACCACATCCAAGGAAGGCAGCAGGCGCGC  359
             ||||||||||||||||||||||||||||||||||||||||||||||||||||||||||||
Sbjct  321   CGATTCCGGAGAGGGAGCCTGAGAAACGGCTACCACATCCAAGGAAGGCAGCAGGCGCGC  380

Query  360   AAATTACCCAATCCTGACACAGGGAGGTAGTGACAATATATAACGATACAGGGCCTTTGG  419
             ||||||||||||||||||||||||||||||||||||||||||||||||||||||||||||
Sbjct  381   AAATTACCCAATCCTGACACAGGGAGGTAGTGACAATATATAACGATACAGGGCCTTTGG  440

Query  420   TCTTGTAATTGGAATGAGTACAATGTAAATACCTTAACGAGGAACAATTGGAGGGCAAGT  479
             ||||||||||||||||||||||||||||||||||||||||||||||||||||||||||||
Sbjct  441   TCTTGTAATTGGAATGAGTACAATGTAAATACCTTAACGAGGAACAATTGGAGGGCAAGT  500

Query  480   CTGGTGCCAGCAGCCGCGGTAATTCCAGCTCCAATAGCGTATATTAAAGTTGTTGCAGTT  539
             ||||||||||||||||||||||||||||||||||||||||||||||||||||||||||||
Sbjct  501   CTGGTGCCAGCAGCCGCGGTAATTCCAGCTCCAATAGCGTATATTAAAGTTGTTGCAGTT  560

Query  540   AAAAAGCTCGTAGTTGAACTTTGGGCCTGGGCGGACGGTCTACCTATGGTAAGCACTGTT  599
             ||||||||||||||||||||||||||||||||||||||||||||||||||||||||||||
Sbjct  561   AAAAAGCTCGTAGTTGAACTTTGGGCCTGGGCGGACGGTCTACCTATGGTAAGCACTGTT  620

Query  600   GCGGCCGGGTCTTTCCTTCTGGCTAGCCCTCGGGCGAACCAGGACGATTACTTTGAGGAA  659
             ||||||||||||||||||||||||||||||||||||||||||||||||||||||||||||
Sbjct  621   GCGGCCGGGTCTTTCCTTCTGGCTAGCCCTCGGGCGAACCAGGACGATTACTTTGAGGAA  680

Query  660   ATTAGAGTGTTCAAAGCAGGCCTTTGCTCGGATATATTAGCATGGAATAATAGAATAGGA  719
             ||||||||||||||||||||||||||||||||||||||||||||||||||||||||||||
Sbjct  681   ATTAGAGTGTTCAAAGCAGGCCTTTGCTCGGATATATTAGCATGGAATAATAGAATAGGA  740

Query  720   CGCATGGTTCTATTTTGTTGGTTTCTAGGACCATCGTAATGATTAATAGGGACGGTCGGG  779
             ||||||||||||||||||||||||||||||||||||||||||||||||||||||||||||
Sbjct  741   CGCATGGTTCTATTTTGTTGGTTTCTAGGACCATCGTAATGATTAATAGGGACGGTCGGG  800

Query  780   GGCATCAGTATTCAGTCGTCAGAGGTGAAATTCTTGGATTGACTGAAGACTAACTACTGC  839
             ||||||||||||||||||||||||||||||||||||||||||||||||||||||||||||
Sbjct  801   GGCATCAGTATTCAGTCGTCAGAGGTGAAATTCTTGGATTGACTGAAGACTAACTACTGC  860

Query  840   GAAAGCATTTGCCAAGGACGTTTTCATTAATCAAGAACGAAAGTTAGGGGATCGAAGATG  899
             ||||||||||||||||||||||||||||||||||||||||||||||||||||||||||||
Sbjct  861   GAAAGCATTTGCCAAGGACGTTTTCATTAATCAAGAACGAAAGTTAGGGGATCGAAGATG  920

Query  900   ATCAGATACCGTCGTAGTCTTAACCATAAACTATGCCGACTAGGGATCGGGTGGTGCTAC  959
             ||||||||||||||||||||||||||||||||||||||||||||||||||||||||||||
Sbjct  921   ATCAGATACCGTCGTAGTCTTAACCATAAACTATGCCGACTAGGGATCGGGTGGTGCTAC  980

Query  960   TTTGCCCACTCGGCACCTTACGAGAAATCAAAGTTTTTGGGTTCTGGGGGGAGTATGGTC  1019
             ||||||||||||||||||||||||||||||||||||||||||||||||||||||||||||
Sbjct  981   TTTGCCCACTCGGCACCTTACGAGAAATCAAAGTTTTTGGGTTCTGGGGGGAGTATGGTC  1040

Query  1020  GCAAGGCTGAAACTTAAAGGAATTGACGGAAGGGCACCACCAGGAGTGGAGCCTGCGGCT  1079
             ||||||||||||||||||||||||||||||||||||||||||||||||||||||||||||
Sbjct  1041  GCAAGGCTGAAACTTAAAGGAATTGACGGAAGGGCACCACCAGGAGTGGAGCCTGCGGCT  1100

Query  1080  TAATTTGACTCAACACGGGGAAACTCACCAGGTCCAGACGTAATAAGGATTGACAAGTTA  1139
             ||||||||||||||||||||||||||||||||||||||||||||||||||||||||||||
Sbjct  1101  TAATTTGACTCAACACGGGGAAACTCACCAGGTCCAGACGTAATAAGGATTGACAAGTTA  1160

Query  1140  GAGACTTCTCTTGATCTTACGGGTGGTGGTGCATGGCCGTTTTTAGTCCTTGGAGTGATT  1199
             ||||||||||||||||||||||||||||||||||||||||||||||||||||||||||||
Sbjct  1161  GAGACTTCTCTTGATCTTACGGGTGGTGGTGCATGGCCGTTTTTAGTCCTTGGAGTGATT  1220

Query  1200  TGTCTGCTTAATTGCGATAACGGACGAGACCTTAACCTGCTAAATAGGGCTGCGAGCATC  1259
             ||||||||||||||||||||||||||||||||||||||||||||||||||||||||||||
Sbjct  1221  TGTCTGCTTAATTGCGATAACGGACGAGACCTTAACCTGCTAAATAGGGCTGCGAGCATC  1280

Query  1260  TGCTCGGGTGCTCTTCTTAGAGGGACTATGGGTATCAAACCCATGGAAGTTTGAGGCAAC  1319
             ||||||||||||||||||||||||||||||||||||||||||||||||||||||||||||
Sbjct  1281  TGCTCGGGTGCTCTTCTTAGAGGGACTATGGGTATCAAACCCATGGAAGTTTGAGGCAAC  1340

Query  1320  AACAGGTCTGTGATGCCCTTAGACGTTCTGGGCCGCACGCGCGCTACACTGACGGAGCCA  1379
             ||||||||||||||||||||||||||||||||||||||||||||||||||||||||||||
Sbjct  1341  AACAGGTCTGTGATGCCCTTAGACGTTCTGGGCCGCACGCGCGCTACACTGACGGAGCCA  1400

Query  1380  GCAAGTCCAACCTTGGTCGAGAGGCCCGGGTAATCTCGTGAAACTCCGTCGTGCTGGGGA  1439
             ||||||||||||||||||||||||||||||||||||||||||||||||||||||||||||
Sbjct  1401  GCAAGTCCAACCTTGGTCGAGAGGCCCGGGTAATCTCGTGAAACTCCGTCGTGCTGGGGA  1460

Query  1440  TAGAGCATTGTAATTTTTGCTCTTCAACGAGGAATTCCTAGTAAGCGCAAGTCATCAGCT  1499
             ||||||||||||||||||||||||||||||||||||||||||||||||||||||||||||
Sbjct  1461  TAGAGCATTGTAATTTTTGCTCTTCAACGAGGAATTCCTAGTAAGCGCAAGTCATCAGCT  1520

Query  1500  TGCGTTGATTACGTCCCTGCCCTTTGTACACACCGCCCGTCGCTACTACCGATTGAATGG  1559
             ||||||||||||||||||||||||||||||||||||||||||||||||||||||||||||
Sbjct  1521  TGCGTTGATTACGTCCCTGCCCTTTGTACACACCGCCCGTCGCTACTACCGATTGAATGG  1580

Query  1560  CTTAGTGAGGCTTCAAGATTGGCGCCGCGGGAGGGGCAACTTTCCCATGGGGCCGAGAAT  1619
             ||||||||||||||||||||||||||||||||||||||||||||||||||||||||||||
Sbjct  1581  CTTAGTGAGGCTTCAAGATTGGCGCCGCGGGAGGGGCAACTTTCCCATGGGGCCGAGAAT  1640

Query  1620  CTAGTCAAACTTGGTCATTA  1639
             ||||||||||||||||||||
Sbjct  1641  CTAGTCAAACTTGGTCATTA  1660
```

Pichia kudriavzevii 18S ribosomal RNA (C5L36_RDN18a), rRNA

Sequence ID: **XR_003834612.1** Length: 1766 Number of Matches: 1

See 2 more title(s)

Range 1: 70 to 1708

**Score**	**Expect**	**Identities**	**Gaps**	**Strand**	**Frame**
3020 bits(1635)	0.0()	1638/1639(99%)	1/1639(0%)	Plus/Plus	

Features:

```
Query  1     CATTATACGGTG-AACTGCGAATGGCTCATTAAATCAGTTATCGTTTATTTGATAGTTCC  59
             |||||||||||| |||||||||||||||||||||||||||||||||||||||||||||||
Sbjct  70    CATTATACGGTGAAACTGCGAATGGCTCATTAAATCAGTTATCGTTTATTTGATAGTTCC  129

Query  60    GTTCTACATGGATAACCGTGGAAAATCTAGAGCTAATACATGCGTAAAGCCCCGACTTCG  119
             ||||||||||||||||||||||||||||||||||||||||||||||||||||||||||||
Sbjct  130   GTTCTACATGGATAACCGTGGAAAATCTAGAGCTAATACATGCGTAAAGCCCCGACTTCG  189

Query  120   GGAGGGGTGTATTTATTAGATAAAAAATCAATGCCCTCGGGCCTTTTGATGATTCATAAT  179
             ||||||||||||||||||||||||||||||||||||||||||||||||||||||||||||
Sbjct  190   GGAGGGGTGTATTTATTAGATAAAAAATCAATGCCCTCGGGCCTTTTGATGATTCATAAT  249

Query  180   AACTTTTCGAAGCTCATGGCCTTGCGCCGGAGCTGGTTCATTCAAATTTCTGCCCTATCA  239
             ||||||||||||||||||||||||||||||||||||||||||||||||||||||||||||
Sbjct  250   AACTTTTCGAAGCTCATGGCCTTGCGCCGGAGCTGGTTCATTCAAATTTCTGCCCTATCA  309

Query  240   ACTTTCGATGGTAGGATAGAGGCCTACCATGGTTTTCACGGGTAACGGGGAATAAGGGTT  299
             ||||||||||||||||||||||||||||||||||||||||||||||||||||||||||||
Sbjct  310   ACTTTCGATGGTAGGATAGAGGCCTACCATGGTTTTCACGGGTAACGGGGAATAAGGGTT  369

Query  300   CGATTCCGGAGAGGGAGCCTGAGAAACGGCTACCACATCCAAGGAAGGCAGCAGGCGCGC  359
             ||||||||||||||||||||||||||||||||||||||||||||||||||||||||||||
Sbjct  370   CGATTCCGGAGAGGGAGCCTGAGAAACGGCTACCACATCCAAGGAAGGCAGCAGGCGCGC  429

Query  360   AAATTACCCAATCCTGACACAGGGAGGTAGTGACAATATATAACGATACAGGGCCTTTGG  419
             ||||||||||||||||||||||||||||||||||||||||||||||||||||||||||||
Sbjct  430   AAATTACCCAATCCTGACACAGGGAGGTAGTGACAATATATAACGATACAGGGCCTTTGG  489

Query  420   TCTTGTAATTGGAATGAGTACAATGTAAATACCTTAACGAGGAACAATTGGAGGGCAAGT  479
             ||||||||||||||||||||||||||||||||||||||||||||||||||||||||||||
Sbjct  490   TCTTGTAATTGGAATGAGTACAATGTAAATACCTTAACGAGGAACAATTGGAGGGCAAGT  549

Query  480   CTGGTGCCAGCAGCCGCGGTAATTCCAGCTCCAATAGCGTATATTAAAGTTGTTGCAGTT  539
             ||||||||||||||||||||||||||||||||||||||||||||||||||||||||||||
Sbjct  550   CTGGTGCCAGCAGCCGCGGTAATTCCAGCTCCAATAGCGTATATTAAAGTTGTTGCAGTT  609

Query  540   AAAAAGCTCGTAGTTGAACTTTGGGCCTGGGCGGACGGTCTACCTATGGTAAGCACTGTT  599
             ||||||||||||||||||||||||||||||||||||||||||||||||||||||||||||
Sbjct  610   AAAAAGCTCGTAGTTGAACTTTGGGCCTGGGCGGACGGTCTACCTATGGTAAGCACTGTT  669

Query  600   GCGGCCGGGTCTTTCCTTCTGGCTAGCCCTCGGGCGAACCAGGACGATTACTTTGAGGAA  659
             ||||||||||||||||||||||||||||||||||||||||||||||||||||||||||||
Sbjct  670   GCGGCCGGGTCTTTCCTTCTGGCTAGCCCTCGGGCGAACCAGGACGATTACTTTGAGGAA  729

Query  660   ATTAGAGTGTTCAAAGCAGGCCTTTGCTCGGATATATTAGCATGGAATAATAGAATAGGA  719
             ||||||||||||||||||||||||||||||||||||||||||||||||||||||||||||
Sbjct  730   ATTAGAGTGTTCAAAGCAGGCCTTTGCTCGGATATATTAGCATGGAATAATAGAATAGGA  789

Query  720   CGCATGGTTCTATTTTGTTGGTTTCTAGGACCATCGTAATGATTAATAGGGACGGTCGGG  779
             ||||||||||||||||||||||||||||||||||||||||||||||||||||||||||||
Sbjct  790   CGCATGGTTCTATTTTGTTGGTTTCTAGGACCATCGTAATGATTAATAGGGACGGTCGGG  849

Query  780   GGCATCAGTATTCAGTCGTCAGAGGTGAAATTCTTGGATTGACTGAAGACTAACTACTGC  839
             ||||||||||||||||||||||||||||||||||||||||||||||||||||||||||||
Sbjct  850   GGCATCAGTATTCAGTCGTCAGAGGTGAAATTCTTGGATTGACTGAAGACTAACTACTGC  909

Query  840   GAAAGCATTTGCCAAGGACGTTTTCATTAATCAAGAACGAAAGTTAGGGGATCGAAGATG  899
             ||||||||||||||||||||||||||||||||||||||||||||||||||||||||||||
Sbjct  910   GAAAGCATTTGCCAAGGACGTTTTCATTAATCAAGAACGAAAGTTAGGGGATCGAAGATG  969

Query  900   ATCAGATACCGTCGTAGTCTTAACCATAAACTATGCCGACTAGGGATCGGGTGGTGCTAC  959
             ||||||||||||||||||||||||||||||||||||||||||||||||||||||||||||
Sbjct  970   ATCAGATACCGTCGTAGTCTTAACCATAAACTATGCCGACTAGGGATCGGGTGGTGCTAC  1029

Query  960   TTTGCCCACTCGGCACCTTACGAGAAATCAAAGTTTTTGGGTTCTGGGGGGAGTATGGTC  1019
             ||||||||||||||||||||||||||||||||||||||||||||||||||||||||||||
Sbjct  1030  TTTGCCCACTCGGCACCTTACGAGAAATCAAAGTTTTTGGGTTCTGGGGGGAGTATGGTC  1089

Query  1020  GCAAGGCTGAAACTTAAAGGAATTGACGGAAGGGCACCACCAGGAGTGGAGCCTGCGGCT  1079
             ||||||||||||||||||||||||||||||||||||||||||||||||||||||||||||
Sbjct  1090  GCAAGGCTGAAACTTAAAGGAATTGACGGAAGGGCACCACCAGGAGTGGAGCCTGCGGCT  1149

Query  1080  TAATTTGACTCAACACGGGGAAACTCACCAGGTCCAGACGTAATAAGGATTGACAAGTTA  1139
             ||||||||||||||||||||||||||||||||||||||||||||||||||||||||||||
Sbjct  1150  TAATTTGACTCAACACGGGGAAACTCACCAGGTCCAGACGTAATAAGGATTGACAAGTTA  1209

Query  1140  GAGACTTCTCTTGATCTTACGGGTGGTGGTGCATGGCCGTTTTTAGTCCTTGGAGTGATT  1199
             ||||||||||||||||||||||||||||||||||||||||||||||||||||||||||||
Sbjct  1210  GAGACTTCTCTTGATCTTACGGGTGGTGGTGCATGGCCGTTTTTAGTCCTTGGAGTGATT  1269

Query  1200  TGTCTGCTTAATTGCGATAACGGACGAGACCTTAACCTGCTAAATAGGGCTGCGAGCATC  1259
             ||||||||||||||||||||||||||||||||||||||||||||||||||||||||||||
Sbjct  1270  TGTCTGCTTAATTGCGATAACGGACGAGACCTTAACCTGCTAAATAGGGCTGCGAGCATC  1329

Query  1260  TGCTCGGGTGCTCTTCTTAGAGGGACTATGGGTATCAAACCCATGGAAGTTTGAGGCAAC  1319
             ||||||||||||||||||||||||||||||||||||||||||||||||||||||||||||
Sbjct  1330  TGCTCGGGTGCTCTTCTTAGAGGGACTATGGGTATCAAACCCATGGAAGTTTGAGGCAAC  1389

Query  1320  AACAGGTCTGTGATGCCCTTAGACGTTCTGGGCCGCACGCGCGCTACACTGACGGAGCCA  1379
             ||||||||||||||||||||||||||||||||||||||||||||||||||||||||||||
Sbjct  1390  AACAGGTCTGTGATGCCCTTAGACGTTCTGGGCCGCACGCGCGCTACACTGACGGAGCCA  1449

Query  1380  GCAAGTCCAACCTTGGTCGAGAGGCCCGGGTAATCTCGTGAAACTCCGTCGTGCTGGGGA  1439
             ||||||||||||||||||||||||||||||||||||||||||||||||||||||||||||
Sbjct  1450  GCAAGTCCAACCTTGGTCGAGAGGCCCGGGTAATCTCGTGAAACTCCGTCGTGCTGGGGA  1509

Query  1440  TAGAGCATTGTAATTTTTGCTCTTCAACGAGGAATTCCTAGTAAGCGCAAGTCATCAGCT  1499
             ||||||||||||||||||||||||||||||||||||||||||||||||||||||||||||
Sbjct  1510  TAGAGCATTGTAATTTTTGCTCTTCAACGAGGAATTCCTAGTAAGCGCAAGTCATCAGCT  1569

Query  1500  TGCGTTGATTACGTCCCTGCCCTTTGTACACACCGCCCGTCGCTACTACCGATTGAATGG  1559
```

```
Sbjct  1570   TGCGTTGATTACGTCCCTGCCCTTTGTACACACCGCCCGTCGCTACTACCGATTGAATGG  1629
Query  1560   CTTAGTGAGGCTTCAAGATTGGCGCCGCGGGAGGGGCAACTTTCCCATGGGGCCGAGAAT  1619
Sbjct  1630   CTTAGTGAGGCTTCAAGATTGGCGCCGCGGGAGGGGCAACTTTCCCATGGGGCCGAGAAT  1689
Query  1620   CTAGTCAAACTTGGTCATT  1638
Sbjct  1690   CTAGTCAAACTTGGTCATT  1708
```

Pichia kudriavzevii strain CY902 chromosome III

Sequence ID: **CP039617.1** Length: 2577713 Number of Matches: 2

Range 1: 2554217 to 2555855

Score	Expect	Identities	Gaps	Strand	Frame
3020 bits(1635)	0.0()	1638/1639(99%)	1/1639(0%)	Plus/Plus	

Features:

```
Query  1        CATTATACGGTG-AACTGCGAATGGCTCATTAAATCAGTTATCGTTTATTTGATAGTTCC  59
Sbjct  2554217  CATTATACGGTGAAACTGCGAATGGCTCATTAAATCAGTTATCGTTTATTTGATAGTTCC  2554276
Query  60       GTTCTACATGGATAACCGTGGAAAATCTAGAGCTAATACATGCGTAAAGCCCCGACTTCG  119
Sbjct  2554277  GTTCTACATGGATAACCGTGGAAAATCTAGAGCTAATACATGCGTAAAGCCCCGACTTCG  2554336
Query  120      GGAGGGGTGTATTTATTAGATAAAAAATCAATGCCCTCGGGCCTTTTGATGATTCATAAT  179
Sbjct  2554337  GGAGGGGTGTATTTATTAGATAAAAAATCAATGCCCTCGGGCCTTTTGATGATTCATAAT  2554396
Query  180      AACTTTTCGAAGCTCATGGCCTTGCGCCGGAGCTGGTTCATTCAAATTTCTGCCCTATCA  239
Sbjct  2554397  AACTTTTCGAAGCTCATGGCCTTGCGCCGGAGCTGGTTCATTCAAATTTCTGCCCTATCA  2554456
Query  240      ACTTTCGATGGTAGGATAGAGGCCTACCATGGTTTTCACGGGTAACGGGGAATAAGGGTT  299
Sbjct  2554457  ACTTTCGATGGTAGGATAGAGGCCTACCATGGTTTTCACGGGTAACGGGGAATAAGGGTT  2554516
Query  300      CGATTCCGGAGAGGGAGCCTGAGAAACGGCTACCACATCCAAGGAAGGCAGCAGGCGCGC  359
Sbjct  2554517  CGATTCCGGAGAGGGAGCCTGAGAAACGGCTACCACATCCAAGGAAGGCAGCAGGCGCGC  2554576
Query  360      AAATTACCCAATCCTGACACAGGGAGGTAGTGACAATATATAACGATACAGGGCCTTTGG  419
Sbjct  2554577  AAATTACCCAATCCTGACACAGGGAGGTAGTGACAATATATAACGATACAGGGCCTTTGG  2554636
Query  420      TCTTGTAATTGGAATGAGTACAATGTAAATACCTTAACGAGGAACAATTGGAGGGCAAGT  479
Sbjct  2554637  TCTTGTAATTGGAATGAGTACAATGTAAATACCTTAACGAGGAACAATTGGAGGGCAAGT  2554696
Query  480      CTGGTGCCAGCAGCCGCGGTAATTCCAGCTCCAATAGCGTATATTAAAGTTGTTGCAGTT  539
Sbjct  2554697  CTGGTGCCAGCAGCCGCGGTAATTCCAGCTCCAATAGCGTATATTAAAGTTGTTGCAGTT  2554756
Query  540      AAAAAGCTCGTAGTTGAACTTTGGGCCTGGGCGGACGGTCTACCTATGGTAAGCACTGTT  599
Sbjct  2554757  AAAAAGCTCGTAGTTGAACTTTGGGCCTGGGCGGACGGTCTACCTATGGTAAGCACTGTT  2554816
Query  600      GCGGCCGGGTCTTTCCTTCTGGCTAGCCCTCGGGCGAACCAGGACGATTACTTTGAGGAA  659
Sbjct  2554817  GCGGCCGGGTCTTTCCTTCTGGCTAGCCCTCGGGCGAACCAGGACGATTACTTTGAGGAA  2554876
Query  660      ATTAGAGTGTTCAAAGCAGGCCTTTGCTCGGATATATTAGCATGGAATAATAGAATAGGA  719
Sbjct  2554877  ATTAGAGTGTTCAAAGCAGGCCTTTGCTCGGATATATTAGCATGGAATAATAGAATAGGA  2554936
Query  720      CGCATGGTTCTATTTTGTTGGTTTCTAGGACCATCGTAATGATTAATAGGGACGGTCGGG  779
Sbjct  2554937  CGCATGGTTCTATTTTGTTGGTTTCTAGGACCATCGTAATGATTAATAGGGACGGTCGGG  2554996
Query  780      GGCATCAGTATTCAGTCGTCAGAGGTGAAATTCTTGGATTGACTGAAGACTAACTACTGC  839
Sbjct  2554997  GGCATCAGTATTCAGTCGTCAGAGGTGAAATTCTTGGATTGACTGAAGACTAACTACTGC  2555056
Query  840      GAAAGCATTTGCCAAGGACGTTTTCATTAATCAAGAACGAAAGTTAGGGGATCGAAGATG  899
Sbjct  2555057  GAAAGCATTTGCCAAGGACGTTTTCATTAATCAAGAACGAAAGTTAGGGGATCGAAGATG  2555116
Query  900      ATCAGATACCGTCGTAGTCTTAACCATAAACTATGCCGACTAGGGATCGGGTGGTGCTAC  959
Sbjct  2555117  ATCAGATACCGTCGTAGTCTTAACCATAAACTATGCCGACTAGGGATCGGGTGGTGCTAC  2555176
Query  960      TTTGCCCACTCGGCACCTTACGAGAAATCAAAGTTTTTGGGTTCTGGGGGGAGTATGGTC  1019
Sbjct  2555177  TTTGCCCACTCGGCACCTTACGAGAAATCAAAGTTTTTGGGTTCTGGGGGGAGTATGGTC  2555236
Query  1020     GCAAGGCTGAAACTTAAAGGAATTGACGGAAGGGCACCACCAGGAGTGGAGCCTGCGGCT  1079
Sbjct  2555237  GCAAGGCTGAAACTTAAAGGAATTGACGGAAGGGCACCACCAGGAGTGGAGCCTGCGGCT  2555296
Query  1080     TAATTTGACTCAACACGGGGAAACTCACCAGGTCCAGACGTAATAAGGATTGACAAGTTA  1139
Sbjct  2555297  TAATTTGACTCAACACGGGGAAACTCACCAGGTCCAGACGTAATAAGGATTGACAAGTTA  2555356
Query  1140     GAGACTTCTCTTGATCTTACGGGTGGTGGTGCATGGCCGTTTTTAGTCCTTGGAGTGATT  1199
Sbjct  2555357  GAGACTTCTCTTGATCTTACGGGTGGTGGTGCATGGCCGTTTTTAGTCCTTGGAGTGATT  2555416
Query  1200     TGTCTGCTTAATTGCGATAACGGACGAGACCTTAACCTGCTAAATAGGGCTGCGAGCATC  1259
Sbjct  2555417  TGTCTGCTTAATTGCGATAACGGACGAGACCTTAACCTGCTAAATAGGGCTGCGAGCATC  2555476
Query  1260     TGCTCGGGTGCTCTTCTTAGAGGGACTATGGGTATCAAACCCATGGAAGTTTGAGGCAAC  1319
```

```
                ||||||||||||||||||||||||||||||||||||||||||||||||||||||||||||
Sbjct  2555477  TGCTCGGGTGCTCTTCTTAGAGGGACTATGGGTATCAAACCCATGGAAGTTTGAGGCAAC  2555536
Query  1320     AACAGGTCTGTGATGCCCTTAGACGTTCTGGGCCGCACGCGCGCTACACTGACGGAGCCA  1379
                ||||||||||||||||||||||||||||||||||||||||||||||||||||||||||||
Sbjct  2555537  AACAGGTCTGTGATGCCCTTAGACGTTCTGGGCCGCACGCGCGCTACACTGACGGAGCCA  2555596
Query  1380     GCAAGTCCAACCTTGGTCGAGAGGCCCGGGTAATCTCGTGAAACTCCGTCGTGCTGGGGA  1439
                ||||||||||||||||||||||||||||||||||||||||||||||||||||||||||||
Sbjct  2555597  GCAAGTCCAACCTTGGTCGAGAGGCCCGGGTAATCTCGTGAAACTCCGTCGTGCTGGGGA  2555656
Query  1440     TAGAGCATTGTAATTTTTGCTCTTCAACGAGGAATTCCTAGTAAGCGCAAGTCATCAGCT  1499
                ||||||||||||||||||||||||||||||||||||||||||||||||||||||||||||
Sbjct  2555657  TAGAGCATTGTAATTTTTGCTCTTCAACGAGGAATTCCTAGTAAGCGCAAGTCATCAGCT  2555716
Query  1500     TGCGTTGATTACGTCCCTGCCCTTTGTACACACCGCCCGTCGCTACTACCGATTGAATGG  1559
                ||||||||||||||||||||||||||||||||||||||||||||||||||||||||||||
Sbjct  2555717  TGCGTTGATTACGTCCCTGCCCTTTGTACACACCGCCCGTCGCTACTACCGATTGAATGG  2555776
Query  1560     CTTAGTGAGGCTTCAAGATTGGCGCCGCGGGAGGGGCAACTTTCCCATGGGGCCGAGAAT  1619
                ||||||||||||||||||||||||||||||||||||||||||||||||||||||||||||
Sbjct  2555777  CTTAGTGAGGCTTCAAGATTGGCGCCGCGGGAGGGGCAACTTTCCCATGGGGCCGAGAAT  2555836
Query  1620     CTAGTCAAACTTGGTCATT  1638
                |||||||||||||||||||
Sbjct  2555837  CTAGTCAAACTTGGTCATT  2555855
```

Range 2: 2566043 to 2567681

Score	Expect	Identities	Gaps	Strand	Frame
3020 bits(1635)	0.0()	1638/1639(99%)	1/1639(0%)	Plus/Plus	

Features:

```
Query  1        CATTATACGGTG-AACTGCGAATGGCTCATTAAATCAGTTATCGTTTATTTGATAGTTCC  59
                |||||||||||| |||||||||||||||||||||||||||||||||||||||||||||||
Sbjct  2566043  CATTATACGGTGAAACTGCGAATGGCTCATTAAATCAGTTATCGTTTATTTGATAGTTCC  2566102
Query  60       GTTCTACATGGATAACCGTGGAAAATCTAGAGCTAATACATGCGTAAAGCCCCGACTTCG  119
                ||||||||||||||||||||||||||||||||||||||||||||||||||||||||||||
Sbjct  2566103  GTTCTACATGGATAACCGTGGAAAATCTAGAGCTAATACATGCGTAAAGCCCCGACTTCG  2566162
Query  120      GGAGGGGTGTATTTATTAGATAAAAAATCAATGCCCTCGGGCCTTTTGATGATTCATAAT  179
                ||||||||||||||||||||||||||||||||||||||||||||||||||||||||||||
Sbjct  2566163  GGAGGGGTGTATTTATTAGATAAAAAATCAATGCCCTCGGGCCTTTTGATGATTCATAAT  2566222
Query  180      AACTTTTCGAAGCTCATGGCCTTGCGCCGGAGCTGGTTCATTCAAATTTCTGCCCTATCA  239
                ||||||||||||||||||||||||||||||||||||||||||||||||||||||||||||
Sbjct  2566223  AACTTTTCGAAGCTCATGGCCTTGCGCCGGAGCTGGTTCATTCAAATTTCTGCCCTATCA  2566282
Query  240      ACTTTCGATGGTAGGATAGAGGCCTACCATGGTTTTCACGGGTAACGGGGAATAAGGGTT  299
                ||||||||||||||||||||||||||||||||||||||||||||||||||||||||||||
Sbjct  2566283  ACTTTCGATGGTAGGATAGAGGCCTACCATGGTTTTCACGGGTAACGGGGAATAAGGGTT  2566342
Query  300      CGATTCCGGAGAGGGAGCCTGAGAAACGGCTACCACATCCAAGGAAGGCAGCAGGCGCGC  359
                ||||||||||||||||||||||||||||||||||||||||||||||||||||||||||||
Sbjct  2566343  CGATTCCGGAGAGGGAGCCTGAGAAACGGCTACCACATCCAAGGAAGGCAGCAGGCGCGC  2566402
Query  360      AAATTACCCAATCCTGACACAGGGAGGTAGTGACAATATATAACGATACAGGGCCTTTGG  419
                ||||||||||||||||||||||||||||||||||||||||||||||||||||||||||||
Sbjct  2566403  AAATTACCCAATCCTGACACAGGGAGGTAGTGACAATATATAACGATACAGGGCCTTTGG  2566462
Query  420      TCTTGTAATTGGAATGAGTACAATGTAAATACCTTAACGAGGAACAATTGGAGGGCAAGT  479
                ||||||||||||||||||||||||||||||||||||||||||||||||||||||||||||
Sbjct  2566463  TCTTGTAATTGGAATGAGTACAATGTAAATACCTTAACGAGGAACAATTGGAGGGCAAGT  2566522
Query  480      CTGGTGCCAGCAGCCGCGGTAATTCCAGCTCCAATAGCGTATATTAAAGTTGTTGCAGTT  539
                ||||||||||||||||||||||||||||||||||||||||||||||||||||||||||||
Sbjct  2566523  CTGGTGCCAGCAGCCGCGGTAATTCCAGCTCCAATAGCGTATATTAAAGTTGTTGCAGTT  2566582
Query  540      AAAAAGCTCGTAGTTGAACTTTGGGCCTGGGCGGACGGTCTACCTATGGTAAGCACTGTT  599
                ||||||||||||||||||||||||||||||||||||||||||||||||||||||||||||
Sbjct  2566583  AAAAAGCTCGTAGTTGAACTTTGGGCCTGGGCGGACGGTCTACCTATGGTAAGCACTGTT  2566642
Query  600      GCGGCCGGGTCTTTCCTTCTGGCTAGCCCTCGGGCGAACCAGGACGATTACTTTGAGGAA  659
                ||||||||||||||||||||||||||||||||||||||||||||||||||||||||||||
Sbjct  2566643  GCGGCCGGGTCTTTCCTTCTGGCTAGCCCTCGGGCGAACCAGGACGATTACTTTGAGGAA  2566702
Query  660      ATTAGAGTGTTCAAAGCAGGCCTTTGCTCGGATATATTAGCATGGAATAATAGAATAGGA  719
                ||||||||||||||||||||||||||||||||||||||||||||||||||||||||||||
Sbjct  2566703  ATTAGAGTGTTCAAAGCAGGCCTTTGCTCGGATATATTAGCATGGAATAATAGAATAGGA  2566762
Query  720      CGCATGGTTCTATTTTGTTGGTTTCTAGGACCATCGTAATGATTAATAGGGACGGTCGGG  779
                ||||||||||||||||||||||||||||||||||||||||||||||||||||||||||||
Sbjct  2566763  CGCATGGTTCTATTTTGTTGGTTTCTAGGACCATCGTAATGATTAATAGGGACGGTCGGG  2566822
Query  780      GGCATCAGTATTCAGTCGTCAGAGGTGAAATTCTTGGATTGACTGAAGACTAACTACTGC  839
                ||||||||||||||||||||||||||||||||||||||||||||||||||||||||||||
Sbjct  2566823  GGCATCAGTATTCAGTCGTCAGAGGTGAAATTCTTGGATTGACTGAAGACTAACTACTGC  2566882
Query  840      GAAAGCATTTGCCAAGGACGTTTTCATTAATCAAGAACGAAAGTTAGGGGATCGAAGATG  899
                ||||||||||||||||||||||||||||||||||||||||||||||||||||||||||||
Sbjct  2566883  GAAAGCATTTGCCAAGGACGTTTTCATTAATCAAGAACGAAAGTTAGGGGATCGAAGATG  2566942
Query  900      ATCAGATACCGTCGTAGTCTTAACCATAAACTATGCCGACTAGGGATCGGGTGGTGCTAC  959
                ||||||||||||||||||||||||||||||||||||||||||||||||||||||||||||
Sbjct  2566943  ATCAGATACCGTCGTAGTCTTAACCATAAACTATGCCGACTAGGGATCGGGTGGTGCTAC  2567002
Query  960      TTTGCCCACTCGGCACCTTACGAGAAATCAAAGTTTTTGGGTTCTGGGGGGAGTATGGTC  1019
                ||||||||||||||||||||||||||||||||||||||||||||||||||||||||||||
Sbjct  2567003  TTTGCCCACTCGGCACCTTACGAGAAATCAAAGTTTTTGGGTTCTGGGGGGAGTATGGTC  2567062
Query  1020     GCAAGGCTGAAACTTAAAGGAATTGACGGAAGGGCACCACCAGGAGTGGAGCCTGCGGCT  1079
                ||||||||||||||||||||||||||||||||||||||||||||||||||||||||||||
Sbjct  2567063  GCAAGGCTGAAACTTAAAGGAATTGACGGAAGGGCACCACCAGGAGTGGAGCCTGCGGCT  2567122
Query  1080     TAATTTGACTCAACACGGGGAAACTCACCAGGTCCAGACGTAATAAGGATTGACAAGTTA  1139
```

```
Sbjct  2567123  TAATTTGACTCAACACGGGGAAACTCACCAGGTCCAGACGTAATAAGGATTGACAAGTTA  2567182
Query  1140     GAGACTTCTCTTGATCTTACGGGTGGTGGTGCATGGCCGTTTTTAGTCCTTGGAGTGATT  1199
Sbjct  2567183  GAGACTTCTCTTGATCTTACGGGTGGTGGTGCATGGCCGTTTTTAGTCCTTGGAGTGATT  2567242
Query  1200     TGTCTGCTTAATTGCGATAACGGACGAGACCTTAACCTGCTAAATAGGGCTGCGAGCATC  1259
Sbjct  2567243  TGTCTGCTTAATTGCGATAACGGACGAGACCTTAACCTGCTAAATAGGGCTGCGAGCATC  2567302
Query  1260     TGCTCGGGTGCTCTTCTTAGAGGGACTATGGGTATCAAACCCATGGAAGTTTGAGGCAAC  1319
Sbjct  2567303  TGCTCGGGTGCTCTTCTTAGAGGGACTATGGGTATCAAACCCATGGAAGTTTGAGGCAAC  2567362
Query  1320     AACAGGTCTGTGATGCCCTTAGACGTTCTGGGCCGCACGCGCGCTACACTGACGGAGCCA  1379
Sbjct  2567363  AACAGGTCTGTGATGCCCTTAGACGTTCTGGGCCGCACGCGCGCTACACTGACGGAGCCA  2567422
Query  1380     GCAAGTCCAACCTTGGTCGAGAGGCCCGGGTAATCTCGTGAAACTCCGTCGTGCTGGGGA  1439
Sbjct  2567423  GCAAGTCCAACCTTGGTCGAGAGGCCCGGGTAATCTCGTGAAACTCCGTCGTGCTGGGGA  2567482
Query  1440     TAGAGCATTGTAATTTTTGCTCTTCAACGAGGAATTCCTAGTAAGCGCAAGTCATCAGCT  1499
Sbjct  2567483  TAGAGCATTGTAATTTTTGCTCTTCAACGAGGAATTCCTAGTAAGCGCAAGTCATCAGCT  2567542
Query  1500     TGCGTTGATTACGTCCCTGCCCTTTGTACACACCGCCCGTCGCTACTACCGATTGAATGG  1559
Sbjct  2567543  TGCGTTGATTACGTCCCTGCCCTTTGTACACACCGCCCGTCGCTACTACCGATTGAATGG  2567602
Query  1560     CTTAGTGAGGCTTCAAGATTGGCGCCGCGGGAGGGGCAACTTTCCCATGGGGCCGAGAAT  1619
Sbjct  2567603  CTTAGTGAGGCTTCAAGATTGGCGCCGCGGGAGGGGCAACTTTCCCATGGGGCCGAGAAT  2567662
Query  1620     CTAGTCAAACTTGGTCATT  1638
Sbjct  2567663  CTAGTCAAACTTGGTCATT  2567681
```

Pichia kudriavzevii strain CY902 chromosome II

Sequence ID: **CP039615.1** Length: 2770935 Number of Matches: 2

Range 1: 2747439 to 2749077

Score	Expect	Identities	Gaps	Strand	Frame
3020 bits(1635)	0.0()	1638/1639(99%)	1/1639(0%)	Plus/Plus	

Features:

```
Query  1        CATTATACGGTG-AACTGCGAATGGCTCATTAAATCAGTTATCGTTTATTTGATAGTTCC  59
Sbjct  2747439  CATTATACGGTGAAACTGCGAATGGCTCATTAAATCAGTTATCGTTTATTTGATAGTTCC  2747498
Query  60       GTTCTACATGGATAACCGTGGAAAATCTAGAGCTAATACATGCGTAAAGCCCCGACTTCG  119
Sbjct  2747499  GTTCTACATGGATAACCGTGGAAAATCTAGAGCTAATACATGCGTAAAGCCCCGACTTCG  2747558
Query  120      GGAGGGGTGTATTTATTAGATAAAAAATCAATGCCCTCGGGCCTTTTGATGATTCATAAT  179
Sbjct  2747559  GGAGGGGTGTATTTATTAGATAAAAAATCAATGCCCTCGGGCCTTTTGATGATTCATAAT  2747618
Query  180      AACTTTTCGAAGCTCATGGCCTTGCGCCGGAGCTGGTTCATTCAAATTTCTGCCCTATCA  239
Sbjct  2747619  AACTTTTCGAAGCTCATGGCCTTGCGCCGGAGCTGGTTCATTCAAATTTCTGCCCTATCA  2747678
Query  240      ACTTTCGATGGTAGGATAGAGGCCTACCATGGTTTTCACGGGTAACGGGGAATAAGGGTT  299
Sbjct  2747679  ACTTTCGATGGTAGGATAGAGGCCTACCATGGTTTTCACGGGTAACGGGGAATAAGGGTT  2747738
Query  300      CGATTCCGGAGAGGGAGCCTGAGAAACGGCTACCACATCCAAGGAAGGCAGCAGGCGCGC  359
Sbjct  2747739  CGATTCCGGAGAGGGAGCCTGAGAAACGGCTACCACATCCAAGGAAGGCAGCAGGCGCGC  2747798
Query  360      AAATTACCCAATCCTGACACAGGGAGGTAGTGACAATATATAACGATACAGGGCCTTTGG  419
Sbjct  2747799  AAATTACCCAATCCTGACACAGGGAGGTAGTGACAATATATAACGATACAGGGCCTTTGG  2747858
Query  420      TCTTGTAATTGGAATGAGTACAATGTAAATACCTTAACGAGGAACAATTGGAGGGCAAGT  479
Sbjct  2747859  TCTTGTAATTGGAATGAGTACAATGTAAATACCTTAACGAGGAACAATTGGAGGGCAAGT  2747918
Query  480      CTGGTGCCAGCAGCCGCGGTAATTCCAGCTCCAATAGCGTATATTAAAGTTGTTGCAGTT  539
Sbjct  2747919  CTGGTGCCAGCAGCCGCGGTAATTCCAGCTCCAATAGCGTATATTAAAGTTGTTGCAGTT  2747978
Query  540      AAAAAGCTCGTAGTTGAACTTTGGGCCTGGGCGGACGGTCTACCTATGGTAAGCACTGTT  599
Sbjct  2747979  AAAAAGCTCGTAGTTGAACTTTGGGCCTGGGCGGACGGTCTACCTATGGTAAGCACTGTT  2748038
Query  600      GCGGCCGGGTCTTTCCTTCTGGCTAGCCCTCGGGCGAACCAGGACGATTACTTTGAGGAA  659
Sbjct  2748039  GCGGCCGGGTCTTTCCTTCTGGCTAGCCCTCGGGCGAACCAGGACGATTACTTTGAGGAA  2748098
Query  660      ATTAGAGTGTTCAAAGCAGGCCTTTGCTCGGATATATTAGCATGGAATAATAGAATAGGA  719
Sbjct  2748099  ATTAGAGTGTTCAAAGCAGGCCTTTGCTCGGATATATTAGCATGGAATAATAGAATAGGA  2748158
Query  720      CGCATGGTTCTATTTTGTTGGTTTCTAGGACCATCGTAATGATTAATAGGGACGGTCGGG  779
Sbjct  2748159  CGCATGGTTCTATTTTGTTGGTTTCTAGGACCATCGTAATGATTAATAGGGACGGTCGGG  2748218
Query  780      GGCATCAGTATTCAGTCGTCAGAGGTGAAATTCTTGGATTGACTGAAGACTAACTACTGC  839
Sbjct  2748219  GGCATCAGTATTCAGTCGTCAGAGGTGAAATTCTTGGATTGACTGAAGACTAACTACTGC  2748278
Query  840      GAAAGCATTTGCCAAGGACGTTTTCATTAATCAAGAACGAAAGTTAGGGGATCGAAGATG  899
```

```
                 ||||||||||||||||||||||||||||||||||||||||||||||||||||||||||||
Sbjct  2748279   GAAAGCATTTGCCAAGGACGTTTTCATTAATCAAGAACGAAAGTTAGGGGATCGAAGATG  2748338
Query  900       ATCAGATACCGTCGTAGTCTTAACCATAAACTATGCCGACTAGGGATCGGGTGGTGCTAC  959
                 ||||||||||||||||||||||||||||||||||||||||||||||||||||||||||||
Sbjct  2748339   ATCAGATACCGTCGTAGTCTTAACCATAAACTATGCCGACTAGGGATCGGGTGGTGCTAC  2748398
Query  960       TTTGCCCACTCGGCACCTTACGAGAAATCAAAGTTTTTGGGTTCTGGGGGGAGTATGGTC  1019
                 ||||||||||||||||||||||||||||||||||||||||||||||||||||||||||||
Sbjct  2748399   TTTGCCCACTCGGCACCTTACGAGAAATCAAAGTTTTTGGGTTCTGGGGGGAGTATGGTC  2748458
Query  1020      GCAAGGCTGAAACTTAAAGGAATTGACGGAAGGGCACCACCAGGAGTGGAGCCTGCGGCT  1079
                 ||||||||||||||||||||||||||||||||||||||||||||||||||||||||||||
Sbjct  2748459   GCAAGGCTGAAACTTAAAGGAATTGACGGAAGGGCACCACCAGGAGTGGAGCCTGCGGCT  2748518
Query  1080      TAATTTGACTCAACACGGGGAAACTCACCAGGTCCAGACGTAATAAGGATTGACAAGTTA  1139
                 ||||||||||||||||||||||||||||||||||||||||||||||||||||||||||||
Sbjct  2748519   TAATTTGACTCAACACGGGGAAACTCACCAGGTCCAGACGTAATAAGGATTGACAAGTTA  2748578
Query  1140      GAGACTTCTCTTGATCTTACGGGTGGTGGTGCATGGCCGTTTTTAGTCCTTGGAGTGATT  1199
                 ||||||||||||||||||||||||||||||||||||||||||||||||||||||||||||
Sbjct  2748579   GAGACTTCTCTTGATCTTACGGGTGGTGGTGCATGGCCGTTTTTAGTCCTTGGAGTGATT  2748638
Query  1200      TGTCTGCTTAATTGCGATAACGGACGAGACCTTAACCTGCTAAATAGGGCTGCGAGCATC  1259
                 ||||||||||||||||||||||||||||||||||||||||||||||||||||||||||||
Sbjct  2748639   TGTCTGCTTAATTGCGATAACGGACGAGACCTTAACCTGCTAAATAGGGCTGCGAGCATC  2748698
Query  1260      TGCTCGGGTGCTCTTCTTAGAGGGACTATGGGTATCAAACCCATGGAAGTTTGAGGCAAC  1319
                 ||||||||||||||||||||||||||||||||||||||||||||||||||||||||||||
Sbjct  2748699   TGCTCGGGTGCTCTTCTTAGAGGGACTATGGGTATCAAACCCATGGAAGTTTGAGGCAAC  2748758
Query  1320      AACAGGTCTGTGATGCCCTTAGACGTTCTGGGCCGCACGCGCGCTACACTGACGGAGCCA  1379
                 ||||||||||||||||||||||||||||||||||||||||||||||||||||||||||||
Sbjct  2748759   AACAGGTCTGTGATGCCCTTAGACGTTCTGGGCCGCACGCGCGCTACACTGACGGAGCCA  2748818
Query  1380      GCAAGTCCAACCTTGGTCGAGAGGCCCGGGTAATCTCGTGAAACTCCGTCGTGCTGGGGA  1439
                 ||||||||||||||||||||||||||||||||||||||||||||||||||||||||||||
Sbjct  2748819   GCAAGTCCAACCTTGGTCGAGAGGCCCGGGTAATCTCGTGAAACTCCGTCGTGCTGGGGA  2748878
Query  1440      TAGAGCATTGTAATTTTTGCTCTTCAACGAGGAATTCCTAGTAAGCGCAAGTCATCAGCT  1499
                 ||||||||||||||||||||||||||||||||||||||||||||||||||||||||||||
Sbjct  2748879   TAGAGCATTGTAATTTTTGCTCTTCAACGAGGAATTCCTAGTAAGCGCAAGTCATCAGCT  2748938
Query  1500      TGCGTTGATTACGTCCCTGCCCTTTGTACACACCGCCCGTCGCTACTACCGATTGAATGG  1559
                 ||||||||||||||||||||||||||||||||||||||||||||||||||||||||||||
Sbjct  2748939   TGCGTTGATTACGTCCCTGCCCTTTGTACACACCGCCCGTCGCTACTACCGATTGAATGG  2748998
Query  1560      CTTAGTGAGGCTTCAAGATTGGCGCCGCGGGAGGGGCAACTTTCCCATGGGGCCGAGAAT  1619
                 ||||||||||||||||||||||||||||||||||||||||||||||||||||||||||||
Sbjct  2748999   CTTAGTGAGGCTTCAAGATTGGCGCCGCGGGAGGGGCAACTTTCCCATGGGGCCGAGAAT  2749058
Query  1620      CTAGTCAAACTTGGTCATT  1638
                 |||||||||||||||||||
Sbjct  2749059   CTAGTCAAACTTGGTCATT  2749077
```

Range 2: 2759265 to 2760903

Score	Expect	Identities	Gaps	Strand	Frame
3020 bits(1635)	0.0()	1638/1639(99%)	1/1639(0%)	Plus/Plus	

Features:

```
Query  1         CATTATACGGTG-AACTGCGAATGGCTCATTAAATCAGTTATCGTTTATTTGATAGTTCC  59
                 |||||||||||| |||||||||||||||||||||||||||||||||||||||||||||||
Sbjct  2759265   CATTATACGGTGAAACTGCGAATGGCTCATTAAATCAGTTATCGTTTATTTGATAGTTCC  2759324
Query  60        GTTCTACATGGATAACCGTGGAAAATCTAGAGCTAATACATGCGTAAAGCCCCGACTTCG  119
                 ||||||||||||||||||||||||||||||||||||||||||||||||||||||||||||
Sbjct  2759325   GTTCTACATGGATAACCGTGGAAAATCTAGAGCTAATACATGCGTAAAGCCCCGACTTCG  2759384
Query  120       GGAGGGGTGTATTTATTAGATAAAAAATCAATGCCCTCGGGCCTTTTGATGATTCATAAT  179
                 ||||||||||||||||||||||||||||||||||||||||||||||||||||||||||||
Sbjct  2759385   GGAGGGGTGTATTTATTAGATAAAAAATCAATGCCCTCGGGCCTTTTGATGATTCATAAT  2759444
Query  180       AACTTTTCGAAGCTCATGGCCTTGCGCCGGAGCTGGTTCATTCAAATTTCTGCCCTATCA  239
                 ||||||||||||||||||||||||||||||||||||||||||||||||||||||||||||
Sbjct  2759445   AACTTTTCGAAGCTCATGGCCTTGCGCCGGAGCTGGTTCATTCAAATTTCTGCCCTATCA  2759504
Query  240       ACTTTCGATGGTAGGATAGAGGCCTACCATGGTTTTCACGGGTAACGGGGAATAAGGGTT  299
                 ||||||||||||||||||||||||||||||||||||||||||||||||||||||||||||
Sbjct  2759505   ACTTTCGATGGTAGGATAGAGGCCTACCATGGTTTTCACGGGTAACGGGGAATAAGGGTT  2759564
Query  300       CGATTCCGGAGAGGGAGCCTGAGAAACGGCTACCACATCCAAGGAAGGCAGCAGGCGCGC  359
                 ||||||||||||||||||||||||||||||||||||||||||||||||||||||||||||
Sbjct  2759565   CGATTCCGGAGAGGGAGCCTGAGAAACGGCTACCACATCCAAGGAAGGCAGCAGGCGCGC  2759624
Query  360       AAATTACCCAATCCTGACACAGGGAGGTAGTGACAATATATAACGATACAGGGCCTTTGG  419
                 ||||||||||||||||||||||||||||||||||||||||||||||||||||||||||||
Sbjct  2759625   AAATTACCCAATCCTGACACAGGGAGGTAGTGACAATATATAACGATACAGGGCCTTTGG  2759684
Query  420       TCTTGTAATTGGAATGAGTACAATGTAAATACCTTAACGAGGAACAATTGGAGGGCAAGT  479
                 ||||||||||||||||||||||||||||||||||||||||||||||||||||||||||||
Sbjct  2759685   TCTTGTAATTGGAATGAGTACAATGTAAATACCTTAACGAGGAACAATTGGAGGGCAAGT  2759744
Query  480       CTGGTGCCAGCAGCCGCGGTAATTCCAGCTCCAATAGCGTATATTAAAGTTGTTGCAGTT  539
                 ||||||||||||||||||||||||||||||||||||||||||||||||||||||||||||
Sbjct  2759745   CTGGTGCCAGCAGCCGCGGTAATTCCAGCTCCAATAGCGTATATTAAAGTTGTTGCAGTT  2759804
Query  540       AAAAAGCTCGTAGTTGAACTTTGGGCCTGGGCGGACGGTCTACCTATGGTAAGCACTGTT  599
                 ||||||||||||||||||||||||||||||||||||||||||||||||||||||||||||
Sbjct  2759805   AAAAAGCTCGTAGTTGAACTTTGGGCCTGGGCGGACGGTCTACCTATGGTAAGCACTGTT  2759864
Query  600       GCGGCCGGGTCTTTCCTTCTGGCTAGCCCTCGGGCGAACCAGGACGATTACTTTGAGGAA  659
                 ||||||||||||||||||||||||||||||||||||||||||||||||||||||||||||
Sbjct  2759865   GCGGCCGGGTCTTTCCTTCTGGCTAGCCCTCGGGCGAACCAGGACGATTACTTTGAGGAA  2759924
Query  660       ATTAGAGTGTTCAAAGCAGGCCTTTGCTCGGATATATTAGCATGGAATAATAGAATAGGA  719
```

```
                   ||||||||||||||||||||||||||||||||||||||||||||||||||||||||||||
Sbjct  2759925  ATTAGAGTGTTCAAAGCAGGCCTTTGCTCGGATATATTAGCATGGAATAATAGAATAGGA  2759984

Query  720      CGCATGGTTCTATTTTGTTGGTTTCTAGGACCATCGTAATGATTAATAGGGACGGTCGGG  779
                ||||||||||||||||||||||||||||||||||||||||||||||||||||||||||||
Sbjct  2759985  CGCATGGTTCTATTTTGTTGGTTTCTAGGACCATCGTAATGATTAATAGGGACGGTCGGG  2760044

Query  780      GGCATCAGTATTCAGTCGTCAGAGGTGAAATTCTTGGATTGACTGAAGACTAACTACTGC  839
                ||||||||||||||||||||||||||||||||||||||||||||||||||||||||||||
Sbjct  2760045  GGCATCAGTATTCAGTCGTCAGAGGTGAAATTCTTGGATTGACTGAAGACTAACTACTGC  2760104

Query  840      GAAAGCATTTGCCAAGGACGTTTTCATTAATCAAGAACGAAAGTTAGGGGATCGAAGATG  899
                ||||||||||||||||||||||||||||||||||||||||||||||||||||||||||||
Sbjct  2760105  GAAAGCATTTGCCAAGGACGTTTTCATTAATCAAGAACGAAAGTTAGGGGATCGAAGATG  2760164

Query  900      ATCAGATACCGTCGTAGTCTTAACCATAAACTATGCCGACTAGGGATCGGGTGGTGCTAC  959
                ||||||||||||||||||||||||||||||||||||||||||||||||||||||||||||
Sbjct  2760165  ATCAGATACCGTCGTAGTCTTAACCATAAACTATGCCGACTAGGGATCGGGTGGTGCTAC  2760224

Query  960      TTTGCCCACTCGGCACCTTACGAGAAATCAAAGTTTTTGGGTTCTGGGGGGAGTATGGTC  1019
                ||||||||||||||||||||||||||||||||||||||||||||||||||||||||||||
Sbjct  2760225  TTTGCCCACTCGGCACCTTACGAGAAATCAAAGTTTTTGGGTTCTGGGGGGAGTATGGTC  2760284

Query  1020     GCAAGGCTGAAACTTAAAGGAATTGACGGAAGGGCACCACCAGGAGTGGAGCCTGCGGCT  1079
                ||||||||||||||||||||||||||||||||||||||||||||||||||||||||||||
Sbjct  2760285  GCAAGGCTGAAACTTAAAGGAATTGACGGAAGGGCACCACCAGGAGTGGAGCCTGCGGCT  2760344

Query  1080     TAATTTGACTCAACACGGGGAAACTCACCAGGTCCAGACGTAATAAGGATTGACAAGTTA  1139
                ||||||||||||||||||||||||||||||||||||||||||||||||||||||||||||
Sbjct  2760345  TAATTTGACTCAACACGGGGAAACTCACCAGGTCCAGACGTAATAAGGATTGACAAGTTA  2760404

Query  1140     GAGACTTCTCTTGATCTTACGGGTGGTGGTGCATGGCCGTTTTTAGTCCTTGGAGTGATT  1199
                ||||||||||||||||||||||||||||||||||||||||||||||||||||||||||||
Sbjct  2760405  GAGACTTCTCTTGATCTTACGGGTGGTGGTGCATGGCCGTTTTTAGTCCTTGGAGTGATT  2760464

Query  1200     TGTCTGCTTAATTGCGATAACGGACGAGACCTTAACCTGCTAAATAGGGCTGCGAGCATC  1259
                ||||||||||||||||||||||||||||||||||||||||||||||||||||||||||||
Sbjct  2760465  TGTCTGCTTAATTGCGATAACGGACGAGACCTTAACCTGCTAAATAGGGCTGCGAGCATC  2760524

Query  1260     TGCTCGGGTGCTCTTCTTAGAGGGACTATGGGTATCAAACCCATGGAAGTTTGAGGCAAC  1319
                ||||||||||||||||||||||||||||||||||||||||||||||||||||||||||||
Sbjct  2760525  TGCTCGGGTGCTCTTCTTAGAGGGACTATGGGTATCAAACCCATGGAAGTTTGAGGCAAC  2760584

Query  1320     AACAGGTCTGTGATGCCCTTAGACGTTCTGGGCCGCACGCGCGCTACACTGACGGAGCCA  1379
                ||||||||||||||||||||||||||||||||||||||||||||||||||||||||||||
Sbjct  2760585  AACAGGTCTGTGATGCCCTTAGACGTTCTGGGCCGCACGCGCGCTACACTGACGGAGCCA  2760644

Query  1380     GCAAGTCCAACCTTGGTCGAGAGGCCCGGGTAATCTCGTGAAACTCCGTCGTGCTGGGGA  1439
                ||||||||||||||||||||||||||||||||||||||||||||||||||||||||||||
Sbjct  2760645  GCAAGTCCAACCTTGGTCGAGAGGCCCGGGTAATCTCGTGAAACTCCGTCGTGCTGGGGA  2760704

Query  1440     TAGAGCATTGTAATTTTTGCTCTTCAACGAGGAATTCCTAGTAAGCGCAAGTCATCAGCT  1499
                ||||||||||||||||||||||||||||||||||||||||||||||||||||||||||||
Sbjct  2760705  TAGAGCATTGTAATTTTTGCTCTTCAACGAGGAATTCCTAGTAAGCGCAAGTCATCAGCT  2760764

Query  1500     TGCGTTGATTACGTCCCTGCCCTTTGTACACACCGCCCGTCGCTACTACCGATTGAATGG  1559
                ||||||||||||||||||||||||||||||||||||||||||||||||||||||||||||
Sbjct  2760765  TGCGTTGATTACGTCCCTGCCCTTTGTACACACCGCCCGTCGCTACTACCGATTGAATGG  2760824

Query  1560     CTTAGTGAGGCTTCAAGATTGGCGCCCGCGGGAGGGGCAACTTTCCCATGGGGCCGAGAAT  1619
                ||||||||||||||||||||||||||||||||||||||||||||||||||||||||||||
Sbjct  2760825  CTTAGTGAGGCTTCAAGATTGGCGCCCGCGGGAGGGGCAACTTTCCCATGGGGCCGAGAAT  2760884

Query  1620     CTAGTCAAACTTGGTCATT  1638
                |||||||||||||||||||
Sbjct  2760885  CTAGTCAAACTTGGTCATT  2760903
```

BLAST is a registered trademark of the National Library of Medicine

Support center Mailing list YouTube

- National Library Of Medicine
- National Institutes Of Health
- U.S. Department of Health & Human Services
- USA.gov

NCBI
National Center for Biotechnology Information, U.S. National Library of Medicine 8600 Rockville Pike, Bethesda MD, 20894 USA
Policies and Guidelines | Contact

COVID-19 is an emerging, rapidly evolving situation.
Get the latest public health information from CDC: https://www.coronavirus.gov .
Get the latest research from NIH: https://www.nih.gov/coronavirus.
Find NCBI SARS-CoV-2 literature, sequence, and clinical content: https://www.ncbi.nlm.nih.gov/sars-cov-2/.
