# Supplementary figures and images for: Variations of Soybean Meal and Corn Mixed Substrates in Physicochemical Characteristics and Microbiota During Two-Stage Solid-State Fermentation
Source: Front Microbiol. 2021 Aug 17;12:688839. doi: 10.3389/fmicb.2021.688839 (PMC8416090; doi:10.3389/fmicb.2021.688839)

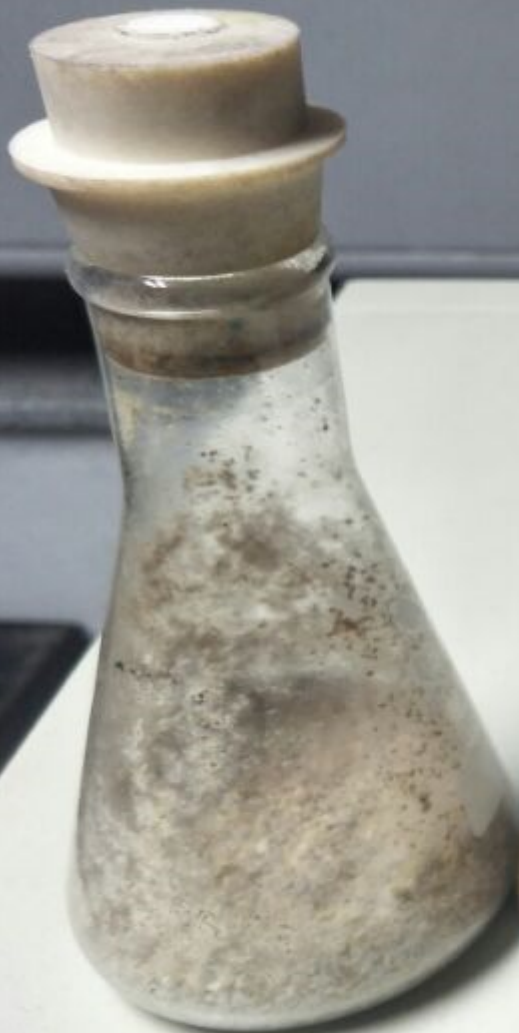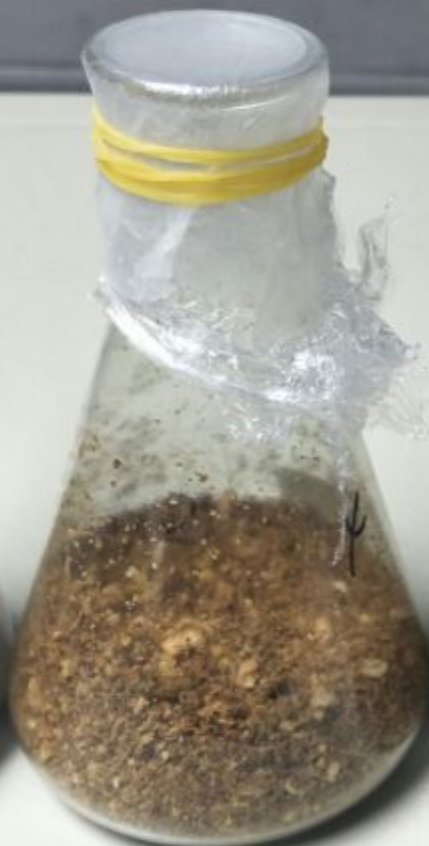

Supplement: Supplementary file 2 [file Data_Sheet_2.PDF]
